# Distal Mutations Shape Substrate-Binding Sites during Evolution of a Metallo-Oxidase into a Laccase

**DOI:** 10.1021/acscatal.2c00336

**Published:** 2022-04-13

**Authors:** Vânia Brissos, Patrícia T. Borges, Reyes Núñez-Franco, Maria Fátima Lucas, Carlos Frazão, Emanuele Monza, Laura Masgrau, Tiago N. Cordeiro, Lígia O. Martins

**Affiliations:** † Instituto de Tecnologia Química e Biológica António Xavier, Universidade Nova de Lisboa, Av da República, 2780-157 Oeiras, Portugal; ‡ Zymvol Biomodeling, Carrer Roc Boronat, 117, 08018 Barcelona, Spain; § Department of Chemistry, Universitat Autònoma de Barcelona, 08193 Bellaterra, Spain

**Keywords:** multicopper oxidases, hyperthermophiles, enzyme specificity, epistasis, enzyme dynamics, allosteric regulation, Aquifex aeolicus

## Abstract

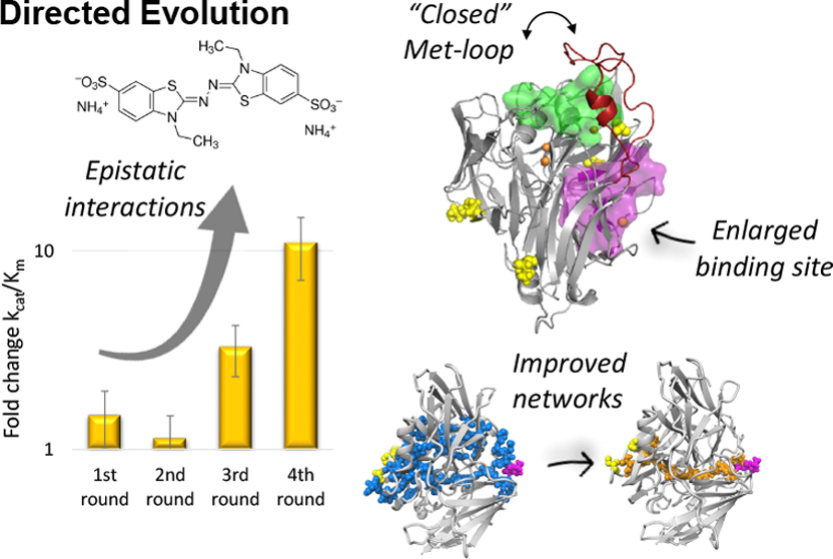

Laccases are in increasing demand as innovative solutions in the biorefinery fields. Here, we combine mutagenesis with structural, kinetic, and *in silico* analyses to characterize the molecular features that cause the evolution of a hyperthermostable metallo-oxidase from the multicopper oxidase family into a laccase (*k*_cat_ 273 s^–1^ for a bulky aromatic substrate). We show that six mutations scattered across the enzyme collectively modulate dynamics to improve the binding and catalysis of a bulky aromatic substrate. The replacement of residues during the early stages of evolution is a stepping stone for altering the shape and size of substrate-binding sites. Binding sites are then fine-tuned through high-order epistasis interactions by inserting distal mutations during later stages of evolution. Allosterically coupled, long-range dynamic networks favor catalytically competent conformational states that are more suitable for recognizing and stabilizing the aromatic substrate. This work provides mechanistic insight into enzymatic and evolutionary molecular mechanisms and spots the importance of iterative experimental and computational analyses to understand local-to-global changes.

## Introduction

Multicopper oxidases (MCOs) are a family of enzymes present in organisms from all three domains of life, archaea, bacteria, and eukarya, which have nearly identical Cu-containing catalytic sites but display a wide range of inorganic and organic substrates as well as catalytic rates. The features and mechanisms that underlie their distinction into two classes, metallo-oxidases and laccases, remain elusive. Laccases can efficiently oxidize many compounds, mainly phenols and aromatic amines, and they have massive potential for biotechnological applications in green chemistry, bioremediation, and biorefinery fields.^1−3^ They are the most promising ligninolytic enzymes and are an environmentally friendly tool for the complete valorization of lignin-derived chemicals, supporting the economic feasibility of the lignocellulose biorefineries.^4^ Metallo-oxidases exhibit high activity for low-valent transient metals such as Cu(I), Fe(II), and Mn(II) and are important in cellular metal homeostasis systems.^5^

MCOs have three Greek key β-barrel cupredoxin domains (domains 1, 2, and 3) that form three spectroscopically different Cu sites: type 1 (T1), type 2 (T2), and the binuclear type 3 (T3).^5,6^ They couple the one-electron oxidation of substrates at the T1 Cu site to the four-electron reduction of molecular oxygen to water at the trinuclear T2/T3 Cu center (TNC). The catalytic mechanism of MCOs involves (i) the reduction of the T1 Cu site, (ii) electron transfer (ET) from the T1 Cu to the TNC *via* a conserved His–Cys–His pathway, and (iii) O_2_ reduction at the TNC.^7^ The broad range of substrates oxidized by MCOs is the result of their noncovalent binding near the T1 His ligands for outer-sphere ET.^7^ The binding site is relatively nonspecific, and a large variety of substrates can be accommodated without being tightly bound.^8,9^ In laccases, the T1 Cu site occupies a depression on the surface of the third domain, allowing one of its histidine ligands to act as a primary electron acceptor. In contrast, the access to the T1 Cu in metallo-oxidases is more spatially constrained, and the His ligands are not easily accessible to reducing substrates. In yeast Fet3P and human ceruloplasmin, two carboxylates are hydrogen-bonded to the His residues of the T1 site, providing an efficient ET pathway.^10,11^ An unusual feature of prokaryotic metallo-oxidases is the occurrence of methionine-rich segments (hereafter termed Met-loops) that partially occlude the T1 Cu.^12−15^ Recently, we characterized the conformational landscape of a 29-residue Met-loop from the *Aquifex aeolicus* McoA metallo-oxidase.^16^ We showed that this segment is a flexible omega-loop that follows open-to-closed transitions with a putative switch-like regulatory function that controls the access of substrates to the T1 Cu site. McoA is encoded within a copper resistance operon and shows remarkable efficiency in the oxidation of cuprous and ferrous ions and has an extreme intrinsic thermostability, a melting temperature of 93 °C, which is worth exploring for biotechnological applications.^17,18^ We had previously engineered this enzyme using directed evolution (DE), and variant 2B3 was identified featuring a 10-fold improved catalytic efficiency for the typical laccase organic substrate, 2,2′-azinobis-(3-ethyl-benzothiazoline-6-sulfonic acid) diammonium salt (ABTS), following insertion of 10 mutations in four rounds of DE.^19^ This variant emerged as a promising biocatalyst for biotechnological applications in industrial settings, considering its thermal robustness and higher turnover numbers for ABTS (*k*_cat_ = 200 s^–1^)^19^ as compared to the fungal laccases from *Trametes versicolor* (*k*_cat_ = 130 s^–1^) and *Trametes hirsuta* (*k*_cat_ = 196 s^–1^)^20^ or the model bacterial CotA-laccase (*k*_cat_ = 144 s^–1^).^21^

In the present study, we studied the optimization of McoA for ABTS oxidation using an integrative experimental and computational strategy. X-ray diffraction, small-angle X-ray scattering (SAXS), Rosetta, and molecular dynamics (MD) measurements show alterations close to the T1 Cu-active site: in the flexibility of short loops and of the long Met-loop that rigidified and explored more closed conformations to favor binding to aromatic substrates, and in the emergence of a new productive binding-cavity widened to facilitate substrate binding. The kinetic analysis of intermediates from the evolutionary trajectory shows that mutations interacting synergistically through epistasis accelerate evolution. The biophysical analysis provided insights into the subtle allosteric interplay of intermolecular networks, dictating enzyme flexibility and conformational selection with effects on alterations of substrate specificity.

## Methods

### Bacterial Strains, Plasmids, and Culture Media

*Escherichia coli* strain DH5α (Novagen) was used for plasmid constructs amplification. *E. coli* Tuner ΔcueO::kan strain, in which the *cueO* gene that codes for the multicopper oxidase CueO was deleted,^22^ was used to express the *mcoA* gene or its evolved variants.^19^ The *mcoA* gene, cloned in the pET-21a (+) plasmid (Novagen), is under the control of the T7 promoter, and its expression is induced by isopropyl β-d-1-thiogalactopyranoside (IPTG). A Luria–Bertani (LB) medium or a terrific broth (TB) medium was used for the maintenance and growth of *E. coli* strains, supplemented with appropriate antibiotics.^19^

### Recombination by DNA Shuffling and Variant Library Construction

The signal peptide present in the N-terminus of McoA was removed from the 2B3 variant following a previously described strategy.^16^ DNA shuffling was performed as reported before.^23,24^ Wild-type and 2B3 variants (without the signal peptide) were amplified by high-fidelity polymerase chain reaction (PCR) using primers pET21D and pET21R (Table S1). An equimolar mixture of each parental gene was digested with 2.5 U mL^–1^ of DNase for 25 min at 15 °C in a thermocycler (MyCycler Thermal Cycler, Biorad) and stopped with ethylenediaminetetraacetic acid (EDTA). The PCR reassembly was carried out in a 20 μL reaction with 3 μL of DNA, 200 μM dNTPs, and 2.5 U of NZYProof polymerase (NZYTech) in NZYProof polymerase buffer. The PCR reassembly products were amplified by high-fidelity PCR in 50 μL reactions with 1 μL of PCR reassembly products, 1 μM primers, 200 μM dNTPs, and 2.5 U of NZYProof polymerase (NZYTech) in NZYProof polymerase buffer, using the conditions previously described for gene amplification.^16^ The PCR products were purified using GFX PCR DNA and a Gel Band Purification kit (GE Healthcare). The final PCR products were digested with *Nde*I/*Eco*RI (Thermofisher), cloned into pET-21a (+) (Novagen), and introduced into electrocompetent *E. coli* Tuner ΔcueO::kan cells.^19^

### Expression of the *mcoA* Variant Library, Cell Disruption, and Activity Screening

Single colonies were picked and cultured in 96-well plates with 200 μL of an LB medium containing ampicillin (100 μg L^–1^) and kanamycin (10 μg L^–1^). Cell cultivation, disruption, and activity screening for ABTS in cell-crude extracts were performed as previously described.^19^ Rescreening of the best variants was enacted to eliminate false positives.

### Construction of Rational-Designed Variants

Single amino acid substitutions in genes coding for wild-type and 2F4 variant or truncated variants in the Met-rich segment (29 residues; segment 327–355) obtained after the removal of internal fragments of variant 2F4 were constructed with the QuickChange site-directed mutagenesis protocol (Stratagene). The following truncated variants were created: loop 5 (with only five residues), lacking residues 329–352 in the Met-loop, loop 7 (lacking residues 330–351), loop 13 (lacking residues 333–348), and loop 19 (lacking residues 336–345). Plasmids containing the genes of interest were used as templates in PCR reactions using appropriate primers (Table S1).^16,19^ The genes were sequenced and verified using the universal T7 primers.

### Production and Purification of Wild-Type McoA and Variants

The cultivation of recombinant *E. coli* Tuner ΔcueO::kan strains in microaerobic conditions for enzyme overproduction and the protein purification protocol was performed as previously described.^19^ The purified protein concentration was determined using the molar absorption coefficient of McoA (ε_280_ = 75 875 M^–1^ cm^–1^; http://web.expasy.org) or the Bradford assay using bovine serum albumin as a standard. *In vitro* copper incorporation was performed by incubating for 30 min in anaerobic conditions, protein preparations as purified with 20 molar equiv of Cu(I) in 20 mM phosphate buffer, 150 mM NaCl, pH 7.4 using freshly prepared [Cu(I)(MeCN)_4_]PF_6_ in argon-purged acetonitrile, by repeated cycles of evacuating/flushing with argon.^25^ Excess copper was removed by washing with metal-free buffer in a centricon unit (Amicon).

### Kinetic Analysis and Stability Assays

At pH 4 and 40 °C, oxidation of ABTS was followed at 420 nm as previously described.^19^ Cuprous and ferrous oxidase activities were estimated at 40 °C by measuring oxygen consumption (Oxygraph; Hansatech).^17^ The kinetic parameters (*K*_m_ and *k*_cat_) were calculated by fitting the results to the Michaelis–Menten equation (Origin software). The kinetic and thermodynamic stability, measuring thermal inactivation, thermal unfolding by differential scanning calorimetry (DSC), and monitoring protein aggregation by static light scattering were performed as previously described.^19^

### Solubility Assays

For protein solubility analysis, 20 μg of soluble and insoluble fractions was loaded onto sodium dodecyl sulfate-polyacrylamide gel electrophoresis (SDS-PAGE). The soluble and insoluble fractions were obtained from the partially purified crude extracts (after heating at 80 °C), and the pellets obtained after lysis were resuspended in lysis buffer. Protein band intensity was measured using Image Lab 4.1, and the solubility was calculated as *I*_S_/(*I*_S_ + *I*_P_) × 100, with *I*_S_ being the intensity of the supernatant band and *I*_P_ being the intensity of the pellet band.

### Crystallization and Data Collection and Processing

Crystallization trials of the 2F4 variant (purified in 20 mM Tris–HCl pH 7.6 supplemented with 200 mM NaCl) were accomplished as for the wild-type McoA enzyme.^16^ Briefly, 2F4 crystals were obtained at 20 °C by the hanging drop vapor-diffusion method in 24-well crystallization plates. Drops of 1 or 2 μL of a protein solution (15 mg mL^–1^) were mixed with 1 μL of the reservoir solution containing 1.5–2.0 M [NH_4_]_2_SO_4_ and equilibrated against 500 μL of the reservoir solution. The best 2F4 crystals appeared after 1 week at 20 °C with 2 M [NH_4_]_2_SO_4_ using 2:1 μL of protein/reservoir solutions, which reached dimensions of 110, 40, and 20 μm. The plate-like crystals showed a blue color. The crystals were transferred to the reservoir solution supplemented with 25% (v/v) glycerol before being flash-cooled in liquid nitrogen. Data sets for 2F4 crystals were collected at the European Synchrotron Radiation Facility (ESRF, Grenoble, France) on beamline ID23-1 with a PILATUS 6M detector 0.9762 Å wavelength radiation, 405 mm crystal-to-detector distance, and 0.10° oscillation widths in a total of 180° rotation for 27 s. Diffraction data were integrated and scaled with XDS.^26^ 2F4 variant crystal data sets were processed in space group *P*2. Data collection details and processing statistics are listed in Table S2.

### Structure Determination and Refinement

A 2F4 variant unit cell contained two molecules in the asymmetric unit, corresponding to a *V*_M_ of 1.99 Å^3^ Da^–1^ and a solvent content of ∼38%.^27,28^ The 2F4 crystal structure was solved by the molecular replacement model using the coordinates of the previously published crystal structure of *A. aeolicus* McoA wild-type (PDB 6SYY)^16^ as the search model. PHASER^29^ within the PHENIX suite^30^ found two molecules in the asymmetric unit, with TFZ values of 8 and 21 indicating a successful structure solution.^31^ The structure was refined with PHENIX.REFINE.^30,32,33^ A set of random intensities (1.5%) from thin resolution shells were selected for cross-validation and optimization of the diffraction *vs* stereochemistry weights. Though refinement comprised standard stereochemistry libraries,^34^ the interatomic distances involving copper centers and their ligands were refined without target restraints. All copper atoms are refined to total occupancies.

The TLSMD server (http://skuld.bmsc.washington.edu/~tlsmd)^35^ defined structural regions of translation, libration, and screw (TLS) refinement. Refinement cycles included atomic coordinates and individual isotropic atomic displacement parameters (a.d.p.s.), TLS refinement, and automatic solvent waters modeling, with hydrogen bonding distances within 2.45–3.40 Å. Refinement cycles were complemented with model comparison against 2*m*|*F*_o_| – *D*|*F*_c_| and *m*|*F*_o_| – *D*|*F*_c_| electron density maps in COOT^36^ to check and improve the fit between the model and electron density maps by adding newly detected solvent ions and molecules from the crystallization medium. This process was repeated iteratively until the *R*_work_ and *R*_free_ converged. The final refinement cycle included all diffraction data. It used the previously optimized weights of stereochemistry *vs* experimental data when the *R*_free_ set was still in use to fine-tune root-mean-square deviation (RMSD) values of bond distances and angles and produce the final structure and *R*_factor_. MOLPROBITY^37^ was used to analyze the stereochemical quality of the system. The accessible surface area (ASA) was calculated with AREAIMOL.^38−40^ The structure figures were prepared using PyMOL.^41,42^ Cavities were determined using Dogsitescorer.^43^ Pairwise distances between MCOs cavities were plotted as a hierarchically clustered heatmap using Seaborn.^44^ The refinement statistics are presented in Table S2. The experimental structure factors and atomic coordinates were available in the Protein Data Bank (www.rcsb.org)^45^ with PDB code 6TTD.

### Small-Angle X-ray Scattering (SAXS)

Size exclusion chromatography (SEC)-SAXS synchrotron data on 2F4 and truncated variant 2F4-loop 5 was measured on the BM29 beamline (ESRF, Grenoble, France) equipped with an in-line high-performance liquid chromatography (HPLC) system (Agilent 1200), injecting 250 μL of samples of 10 mg mL^–1^ into a Superdex 200 10/300 size exclusion column (GE) with a flow rate of 0.5 mL min^–1^, and the mobile phase consisted of 50 mM Tris–HCl, pH 7.6, 150 mM NaCl, and 2 mM tris(2-carboxyethyl)phosphine (TCEP). Samples flowed through the cell chamber at 20 °C, and scattering patterns were stored each second frame with a Pilatus 1M pixel detector without interparticle interactions or aggregation signs and detectable radiation damage. The monomeric elution single-peak region integrated and buffer subtracted the respective scattering intensities to produce the SAXS curves of 2F4 and 2F4-loop 5 using ScÅtter software^46^ and further processed with ATSAS 3.04.^47^ From these SEC-based SAXS curves, we computed the *P*(*r*) distribution function of each sample by indirect Fourier transform spectroscopy. The *R*_g_ values were estimated assuming the Guinier approximation. Moreover, we generated the low-resolution *ab initio* shape of 2F4, with DAMMIF^49^ following the settings and the quality assessment previously used for the wild-type McoA.^16^ SEC-SAXS raw data are available at SASBDB^48^ under the “SAXS studies on hyperthermostable McoA evolved laccase”, with accession code SASDHL8. For more details, see Table S3.

### Met-Loop Ensemble Modeling

As we previously described for wild-type McoA,^16^ we employed state-of-the-art Rosetta loop modeling with SAXS to model the missing methionine-rich loop (Met-loop) and capture its structural plasticity and preferences. In brief, we created 2500 models (the initial pool) and analyzed the Met-loop as subensembles (*N*_se_) that minimize eq 1
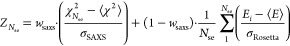
1with the SAXS discrepancy () score given as
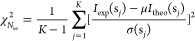
2where *K* is the number of data points of the SEC-SAXS profile (*I*_exp_), σ(*s*_*j*_) is their standard deviation, μ is the scaling factor, and *I*_theo_ is the theoretical SAXS curve obtained by averaging the scattering of the selected models
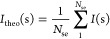
3

For multiple subensembles, we computed the distance distribution of the center-of-mass of the Met-loop to T1 Cu (*d*_Metloop-T1Cu_) and the ASA near the T1 Cu site (ABTS site) and used this metric to categorize the models as open or closed states.

### Homology Modeling

We also created *in silico* loop-truncated models of wild type and 2F4. We modeled the remaining five residues onto the crystal structures of wild type (PDB 6SYY) and 2F4 (PDB 6TTD) with Yasara^50^ that searches a PDB-based database for loop candidates. All parameters had default values. We then used molecular dynamics to sample the best models. Simulations frames were utilized for ensemble docking (more details below).

### Molecular Dynamics

MD simulations of full-length 2F4 and loop-truncated wild-type and 2F4 variants (loop 5) were set up and ran with Yasara^51^ using the AMBER14 force field^52^ and the TIP3P water model.^53^ The general setup included hydrogen bonds’ network optimization, p*K*_a_ prediction at pH 4,^54^ solvation with a 9 Å water buffer to form a cubic box, and neutralization with NaCl. The system’s energy was minimized, with the steepest descendent and simulated annealing, and equilibrated for 5 ns using an integration step of 1 fs. In the first half of equilibration, the system was heated up to 300 K; then, the temperature was fixed (together with the box volume). Production MD runs were carried out, updating the bonded- and nonbonded forces every 2 and 4 fs, respectively. We run four 250 ns independent simulations for each Met-loop-truncated variant (*i.e.*, for wild type and 2F4). For 2F4, three different models of the Met-loop were used as starting structures in the MD simulations, as we previously had done for the wild-type McoA.^16^ They corresponded to a closed and an open conformation of the Met-loop and the best single model generated by the Rosetta/SAXS-based modeling. Two independent simulations of 600 ns were run from each initial structure corresponding to 3.6 μs of simulation. In all cases, temperature control was achieved with the Berendsen thermostat^55^ as implemented in Yasara. The van der Waals forces’ cutoff was set at 8 Å, while long-range electrostatics forces were treated with the particle mesh Ewald algorithm.^56^ LINCS^57^ and SETTLE^58^ were adopted to restrain stretching and bending terms involving hydrogen atoms and water molecules in the system. Principal component analysis (PCA) was used to help identify structural fluctuations along the 2F4 MD trajectories. For that, we built a positional covariance matrix constructed using only the coordinates of Cα atoms extracted from an MD frame every 1.8 ns, with all frames previously aligned to eliminate translational and rotational motions. From the set of eigenvectors and eigenvalues estimated by the matrix diagonalization, the top two ones (principal components 1 and 2, PC1 and PC2) were used for subsequent analysis. Dynamic cross-correlation analysis based on Cα displacements allowed us to detect residues that moved in a correlated or anticorrelated motion in the MD simulations of 2F4 and the previously ran 3.6 μs MD simulation of wild-type McoA. A comparison of two pairwise dynamical cross-correlation (DCC) matrices compared residues with differential dynamical behavior between 2F4 and wild type. VMD version 1.9.3^59^ was used to generate the molecular graphic shown in Figure S14b.

### Ensemble Docking

All docking simulations were set up and run with Yasara and carried out on 100 equally spaced simulation frames from each trajectory (and the protonation state of titrable residues corresponds to the ones at pH 4, as in the MD simulations). ABTS (negatively charged)^60^ was first docked on the complete protein with Vina.^61,62^ This allowed detecting reactive binding modes for ABTS, redocked on a 10 Å cell centered on each identified binding site. Considering that the Met-loop increases the structural diversity of the system, 333 equally spaced simulation frames were used from each trajectory to conduct the ABTS docking. As for the loop-truncated variants, a global docking was carried out first, followed by localized docking at the identified binding sites close to the T1 Cu center. Molecular graphics showing the docking results were created with Yasara.

### Protein Residue Network (PRN)

Protein network analysis (PRN) was used to determine optimal and suboptimal communication pathways within the protein. PRNs were built connecting residues based on their dynamical cross-correlation (DCC). In particular, weighted implementation of optimal and suboptimal paths (WISP)^63^ software was used to analyze the full-length and loop-truncated wild-type and 2F4 MD trajectories. Briefly, a node was defined for each residue center-of-mass. A distance to other residues was determined on the residue-to-residue correlation (the stronger the correlation, the shorter the length). Based on that, WISP can rapidly find the optimal (shortest) and suboptimal pathways between distinct protein residues that potentially define an allosteric communication network. The simulations were analyzed by generating a protein graph with a center-of-mass cutoff distance of 4.5 Å to determine whether two residues were connected nodes in the predicted allosteric path. Up to 15 suboptimal paths were calculated for each residue pair.

### Other Methods

UV–visible absorption spectra in the far-UV region of purified enzymes were obtained as previously described.^19^ Redox titrations were performed as previously described.^17^ The copper content was determined through the trichloroacetic acid/bicinchoninic acid method.^64^

## Results

### DNA-Shuffling Distinguishes Beneficial Mutations

We first deleted the 43-residue N-terminal sequence from variant 2B3, coding for a signal twin-arginine translocation (Tat) peptide, which resulted in a variant without the signal peptide (2B3wsp). 2B3wsp has comparable biochemical and kinetic properties to 2B3 but gives higher yields of the soluble enzyme (Tables S4 and S5 and Figure S1). This led to the removal of two mutations, F17S and V19A, present in the Tat peptide. The mutations did not alter the activity toward ABTS (Table S5). The random recombination of genes coding for the 2B3wsp and the wild type by DNA shuffling allowed the identification of the smallest subset of beneficial mutations that have a functional impact on 2B3wsp. After screening a library of ∼1300 variants for ABTS activity and thermal inactivation at 90 °C, variant 2F4 was selected. 2F4 contains only six mutations (Table S6). It has spectroscopic, kinetic, and stability properties comparable to 2B3wsp (Tables S4 and S5 and Figure S1) and a slight increase in the redox potential (∼11 mV) than that of wild-type McoA (Table S4). The 2F4 variant shows similar kinetic parameters to the wild type for the metal ions Cu(I) and Fe(II) (Table S5).

### X-ray Structure Shows Variations Close to the T1 Cu Center

**Figure 1:**
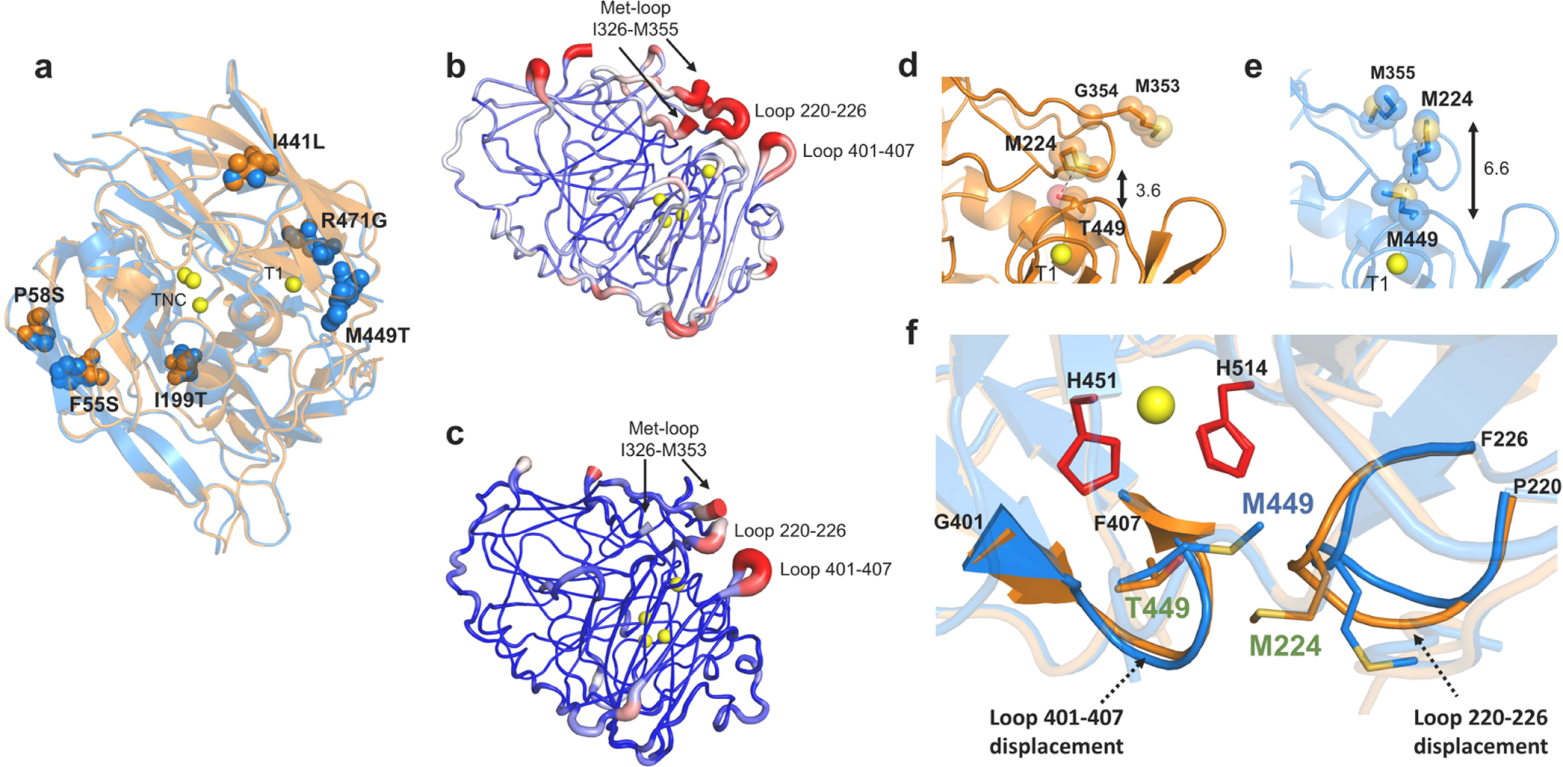
Overall structure and the T1 Cu site environment in 2F4 and wild type. (a) Cartoon representation of the transparent secondary structure of the superimposed wild type (blue) and 2F4 (orange). 2F4 mutations S55, S58, T199, L441, T449, and G471 are displayed as orange spheres and their homologous wild-type side chains as blue spheres. Yellow spheres represent 2F4 copper sites. Cartoon representation of the main-chain 2F4 (b) and wild-type (c) structures with thickness proportional to ⟨a.d.p.⟩ values, color-coded from blue (20 Å^2^) to red (130 Å^2^). The distance between residues 449 and 224 (black dashed line and black arrow) in 2F4 (d) and wild type (e). These residues are shown as sticks and transparent spheres with carbon, oxygen, and sulfur atoms colored orange (blue in the wild type), red, and yellow, respectively. M353 and G354 of 2F4 and M355 of McoA are part of the Met-loop region. (f) Zoomed view of regions 220–226 and 401–407 near T1 Cu in the wild-type (blue) and 2F4 variant (orange). The mutation M449T in 2F4 could have led to a structural displacement of the two loops. The residues 224 and 449 are shown as sticks with carbon, oxygen, and sulfur atoms in orange (blue in the wild type), red, and yellow, respectively. The T1 Cu histidine ligands H451 and H514 are shown as red sticks.

The 2F4 structure obtained at a 1.8 Å resolution is similar to the wild type, with a root-mean-square deviation (RMSD) of 0.39 Å between their Cα positions. However, we observe a difference in the occupancies of their copper sites, which are stoichiometric in 2F4, but in the wild type, occupancy varies between 39 and 65%^16^ (Figures 1a and S2a,b and Table S2). The two structures present variations in the flexibility of their loops neighboring the T1 Cu center, with loop 220–226 in 2F4 showing lower atomic displacement parameters (a.d.p.) than the wild-type homolog, 17.9–63.9 and 32.2–80.5 Å^2^, respectively. This is in contrast to loop 401–407, which shows higher a.d.p. values, 47.3–89.2 and 33.3–83.5 Å^2^, respectively (Figure 1b,c).

The McoA structure contains a methionine-rich unstructured loop (residues 327–355) nearby the active T1 Cu site that is not visible in the electron density maps.^16^ In 2F4, the corresponding loop shows two residues, M353 and G354, in electron density maps at the 1 RMSD contour level, which are not visible in the wild-type maps. This extra electron density could have arisen from mutation M449T, at 6 Å of the T1 Cu, inserted during the first round of evolution (Figure 1d,e). The distance between residues 449 and 224 (loop 220–226) is shorter in 2F4 (3.6 Å) than in the wild type (6.6 Å) and is reflected by a structural rearrangement of the M224 side chain in 2F4 toward the T1 Cu site and away from the protein surface. The displacement of M224 possibly caused M353 and G354 to become less flexible, resulting in the electron density of these residues in 2F4 (Figure 1d). Such conformational changes could reasonably well be the basis for the distinct flexibility of loops 220–226 and 401–407 (Figure 1f).

**Figure 2:**
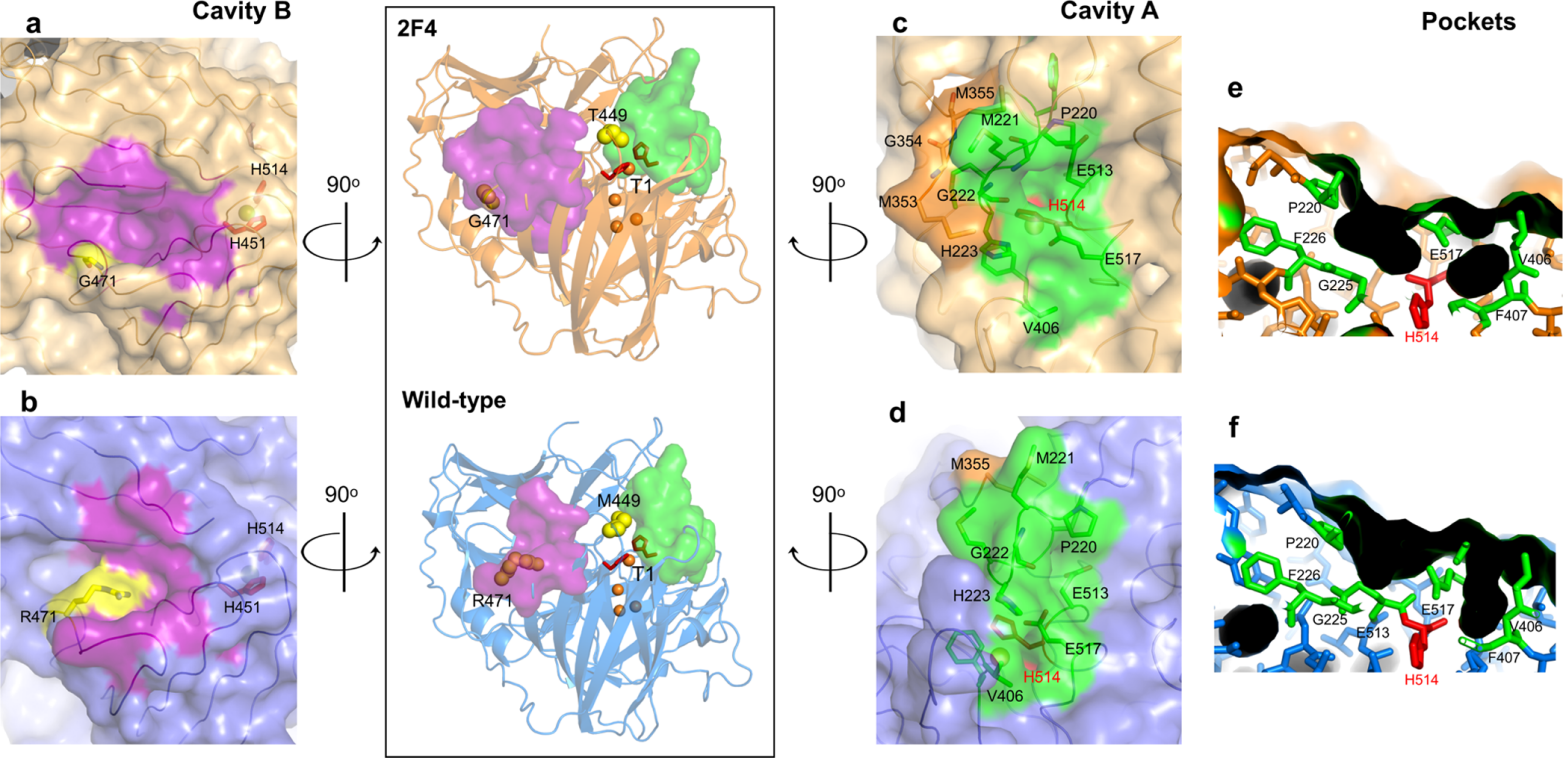
T1 Cu cavities A and B of 2F4 (top) and wild type (bottom). The T1 Cu histidine ligands (H451 and H514) are presented as red sticks. The copper atoms are shown as orange spheres. The mutations M449T and R471G are shown as yellow spheres. Cavities A and B, colored in green and purple, respectively, are shown as ASA. Cavity A shows two small pockets that allow the T1 Cu ligand H514 to access solvent media in 2F4 (c) and wild type (d). Residues part of the Met-rich region with visible electron density (353–355 in 2F4 and 355 in the wild type) are colored in pale orange. (e) and (f) are cutaway views of (c) and (d), respectively, showing pockets in 2F4 and wild type. Cavity B (purple) is located approximately at 180° rotation (in the *y*-axis) apart from cavity A and offers a higher depth in 2F4 (a) relative to wild type (b). The mutated residue 471 is colored yellow.

An accessible surface area (ASA) calculation near the T1 Cu revealed two cavities, A and B, oriented in opposite directions and suitable for substrate binding (Figure 2 and Tables S7 and S8). Cavity A has similar dimensions in both 2F4 and wild-type structures and allows the access of solvent molecules to the partially occluded T1 Cu ligand H514, which shows higher ASA in 2F4 (1.0%) than in McoA (0.2%). The access to H514 is different in the two structures and is partially hindered by the two flexible short loops, 220–226 and 401–407 (Figure 2c–f). The pocket access to H514 is mainly delimited by E517 and P220–G222 of loop 220–226 in 2F4 and by E517 and V406–F407 of loop 401–407 in wild type. The different location of loops facilitates access to the T1 Cu that is more solvent-exposed in the variant structure when compared to the wild type. On the other hand, cavity B is closer to the T1 Cu ligand H451, solvent-occluded in the X-ray crystal structure (Figure 2a,b and Table S8). This cavity shows a significantly higher volume (by ∼300 Å^3^) and surface area (by ∼140 Å^2^) in 2F4 than in the wild type. This can hypothetically be associated with mutation R471G, which replaces a long polar, charged side chain with a slight neutral residue at the entrance to cavity B. This broadening entrance of the active site decreases the high-energy barrier to substrate access and product release. Both cavities are comparable to those in other prokaryotic MCOs (Figure S3): cavity A is closer to the substrate-binding site in *Bacillus subtilis* CotA^9^ and cavity B to the substrate-binding site in *E. coli* CueO and *Thermus thermophilus* Tth multicopper oxidases.^65^

### Allosteric Changes Induce a Met-Loop Closed-Conformation State

**Figure 3:**
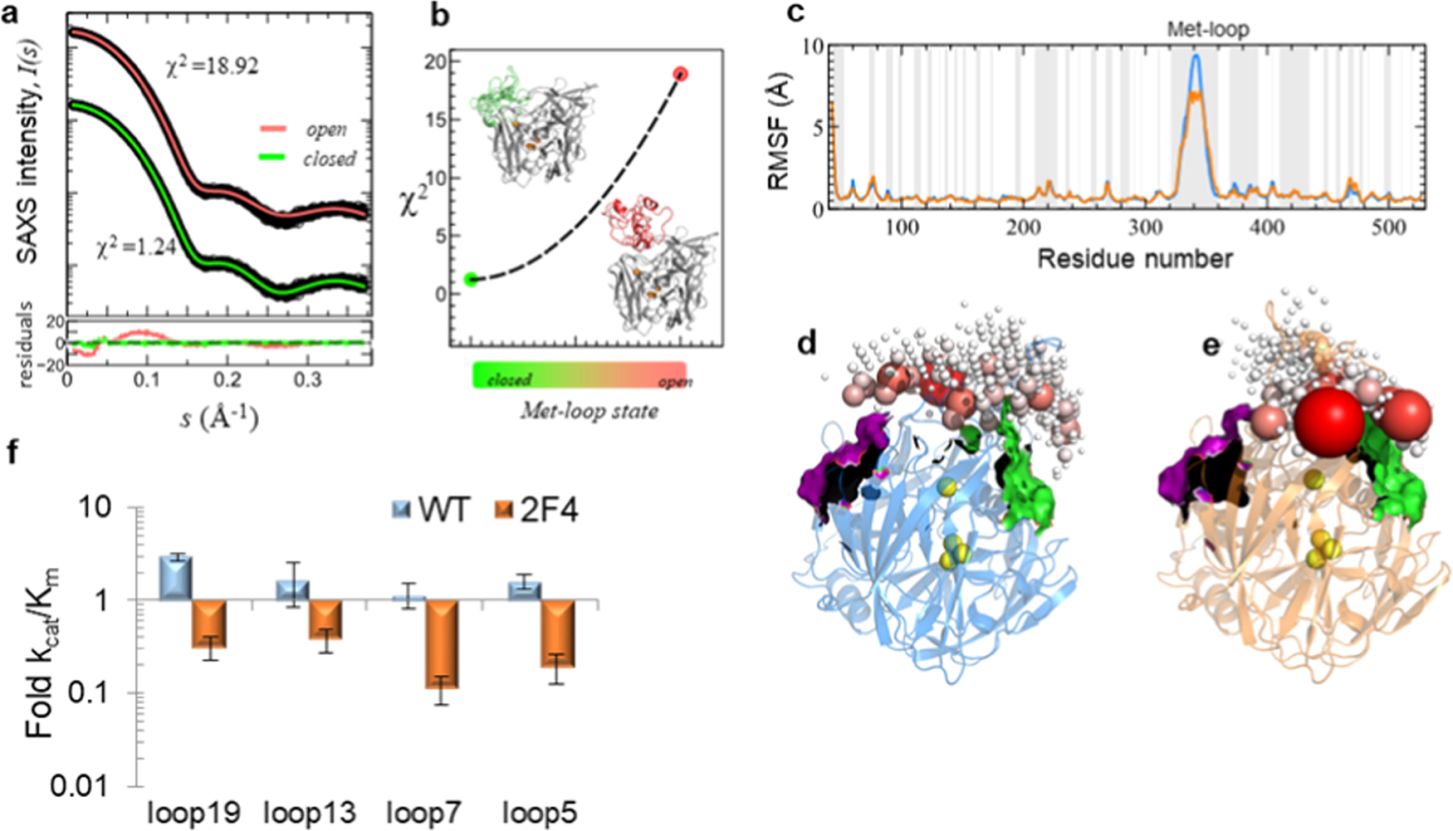
Preferential closed Met-loop in the evolved variant 2F4. (a) Logarithmic-scale representation of scattering intensity, *I*(*s*), as a function of the momentum transfer, *s*, measured for 2F4 (empty black circles). Solid lines are back-calculated curves derived from subensembles of 2F4 with Met-loop in the open state (red) or refined from the ensemble optimization method (EOM) fit of the SAXS data (green). Residuals of absolute values are at the bottom with the same color code. Systematic deviations, away from zero, indicate a poor fit by assuming only open Met-loop structures (χ^2^ = 18.92). The agreement improves by reducing the relative open population, searching best agreement (χ^2^ = 1.24) near 100% of closed models. (b) Plot shows how SAXS discrepancy, χ^2^, varies with the relative population of “closed” (green ribbon) and “open” (red ribbon) states. The best agreement is observed for an ensemble with ∼100% of the closed state, with structures partially preventing access to T1 Cu. For clarity, only five Met-loop structures are represented. (c) Root-mean-square fluctuations (RMSF) for 2F4 (orange) *vs* wild-type McoA (blue) residues over 3.6 μs of simulation. RMSF profiles show that the Met-loop in both variants displays differential mobility, with an apparent reduction in Met-loop flexibility at the T1 Cu interface for 2F4. Loops in McoA/2F4 are shaded in gray. (d, e) Met-loop sampling over the T1 Cu. Wild type and 2F4 are in blue and orange transparent ribbon, and the relative position of the loop tip is displayed as spheres in shades of red. The sphere radius is proportional to the probability of occurrence probability. Cavity A and B are in green and purple surface representation, respectively. The coopers are shown in yellow. (f) Kinetic parameters *k*_cat_/*K*_m_ for the oxidation of ABTS in variants with the Met-rich loop were partially deleted: 2F4 (orange) and wild type (blue). The partial truncation of Met-rich 29-residue loop yielded variants with loops having 19 (loop 19), 13 (loop 13), 7 (loop 7), and 5 (loop 5) residues. Each value averages more than six measurements with standard deviations (bars).

To investigate the conformation of the Met-loop, which is not visible in the electron density maps in the X-ray structure of the 2F4 variant, high-quality SEC-SAXS (Figure S4a) was used to drive the ensemble characterization using Rosetta as a loop model generator.^16^ The resulting SAXS-refined loop ensemble revealed that the Met-loop in 2F4 is flexible, but samples mostly closed states with minimal open structures (Figure 3a,b). We generated 2500 models (initial pool) with the Met-loop distributed 18.4 ± 3.5 Å from the T1 Cu (*d*_Met-loop-T1Cu_) and 243.0 ± 86.8 Å^2^ of ASA near T1 Cu (Figure S5a,b). Upon ensemble optimization against SAXS data, this distribution shifted toward shorter distances and less accessible surfaces, hereafter named the closed state, faithfully enveloped by the SAXS-derived *ab initio* state (Figure S4b). The SAXS curve of the wild type was accurately described by invoking closed and open states in a dynamic equilibrium, with a dominant contribution of Met-loop open-like structures (70 ± 6%).^16^ Conversely, for 2F4, the SEC-SAXS data are compatible with the Met-loop being in the *closed* position (χ^2^ = 1.24) (Figure 3a,b), in line with the apparent smaller overall size when compared to the wild type (Table S3 and Figure S4c). Moreover, we simulated the back-calculated ensembled-based SAXS curve of each state.^16^ Then, by combining both curves weighted by their relative population, we found the best agreement to the experimental SAXS curve of 2F4 at 100% of closed structures (Figure 3b). We further explored this finding using MD simulations (Figures S5c and S6) that suggested that the loop predominantly explores closed states (Figure S6a–c) and that mutations make the Met-loop in 2F4 more rigid (Figure 3c). These observations support the substantial change in the dynamic sampling over T1 Cu compared to the wild type, in which the Met-loop is statistically more open during an equivalent MD (Figure S6d).^16^

The flexible Met-loop is flanked by both cavities A and B and has a role in catalysis, most likely by modulating the access to potential binding sites, hanging and stabilizing substrates near the active site for efficient catalysis. The analysis of the ASA of cavity A or B in the MD trajectories showed that the different Met-loop structures have a more significant impact on cavity A, as it statistically spends more time over this cavity than over cavity B (Figure S7a,b). The conformational loop ensemble reveals that the Met-loop closes over T1 Cu toward loop 220–226, with transitory but predominant contacts with residues of cavity A, which becomes slightly less solvent-exposed in 2F4 (Figure 3d,e). Loop-truncated variants of 2F4 (with loops with 5, 7, 13, and 19 residues, instead of 29) showed a drop in the catalytic efficiency (*k*_cat_/*K*_m_) for ABTS (∼2.6 to 8-fold lower). Still, these variants in the wild-type background offer slightly higher efficiencies (∼1.2 to 3-fold higher *k*_cat_/*K*_m_) (Figure 3f). This indicates a significantly higher beneficial impact of the Met-loop in the interaction with ABTS in the 2F4 variant. The variation in the catalytic efficiency of 2F4 is due to a decrease in the *k*_cat_ and an increase in the *K*_m_ values (Table S9), implying steric changes in the substrate-binding pocket(s) and in the interaction between ABTS and the Met-loop.^16^ It should be noted that the differences in the relative catalytic efficiency between wild type and 2F4 entail that the acquired mutations affect the role of Met-loop in catalysis, revealing high-order epistasis.

### *In Silico* Docking Reveals Catalytically Productive Cavity B

**Figure 4:**
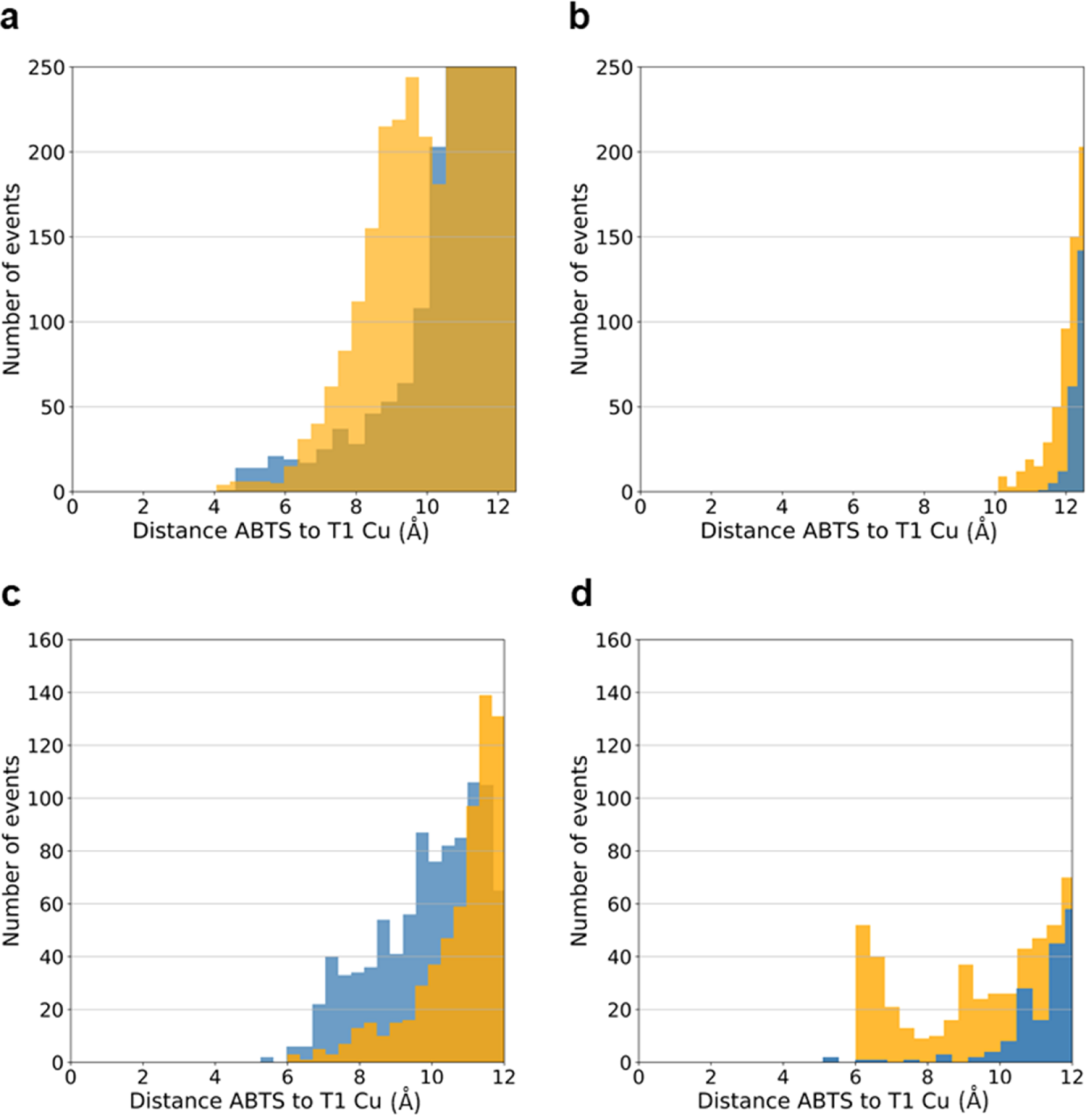
Docking of ABTS to cavity A and cavity B of wild type and 2F4. An ensemble of 1198 and 400 protein structures taken from the MD simulations was used for long-loop (a, b) and truncated-loop (loop 5) (c, d) enzymes, respectively. Histograms show the number of docking solutions as a function of the ABTS distance (Å) to the T1 Cu. Blue and orange colors are used for wild type and 2F4 data, respectively. For long-loop enzymes and cavity A (a), ABTS can get significantly closer to the T1 Cu in 2F4 than the wild type. Also, ABTS binding to cavity B (b) gives slightly shorter ABTS to T1 Cu distances in 2F4. For loop-truncated 2F4, the emergence of a catalytically competent binding site in cavity B is observed (with ABTS to T1 Cu distances of 6.0–7.0 Å). ABTS binding at cavity A is preferred for the truncated wild type, although at slightly longer distances.

ABTS interaction with wild-type McoA and 2F4 was investigated by ensemble docking using snapshots from the MD trajectories. Two main ABTS binding cavities near the T1 Cu were identified corresponding to cavities A and B (Figure S8a,b). The electron donor–acceptor distance is a crucial feature for ET, and therefore the distance to T1 Cu was calculated and binned for all docking poses. For cavity A, distances of ∼12 Å predominate in the wild type. In contrast, two maxima (at ∼9 and 12.5 Å) are present in the 2F4 histogram, with a significantly higher population at <10 Å and stronger binding energies or 2F4 (Figures 4a and S9a,c). Binding to cavity A produces shorter distances (2.5–8.0 Å) of ABTS to H514 than H451 (Figure S9b), consistent with H514 being part of the ET path between the substrate and T1 Cu, as discussed above. For cavity B, docking of ABTS also resulted in shorter ABTS–T1 Cu distances in 2F4 (>10 Å) as compared to the wild type (>11.5 Å) and stronger binding energies (Figures 4b and S9d,f). ABTS is closer to H451 (starting at ∼5/6 Å), suggesting that ET to T1 Cu in this cavity could go through this ligand (Figure S9e). The maximum population is at ∼7.5 and 9.0 Å for 2F4 and wild type, respectively. Altogether, the results agree with the higher catalytic efficiency of 2F4 compared to the wild type.

We ran ABTS dockings for homology models of Met-loop-truncated (loop 5) enzymes to obtain further insight into the role of the Met-loop in 2F4. Two distinct ABTS binding sites (cavities A and B) were observed around the T1 Cu. However, in contrast to what was observed for enzymes bearing the loop, cavity A is found preferentially in the wild type and cavity B in 2F4 (Figures 4c,d and S10). In addition, the wild-type–ABTS binding site is shallow in cavity A, and 2F4 can select cavity B conformations that maximize the probability of ABTS binding close to T1 Cu (Figure S11a,c). For cavity A, in the wild type, shorter ABTS to T1 Cu and H514 distances (∼6.7 and 2.5 Å, respectively) were observed in comparison to 2F4 (∼7.5 and 4 Å), but electron transfer involving H514 is still feasible (Figures 4c and S11a,b). Binding of ABTS to cavity B in 2F4 results in shorter ABTS to T1 Cu distances (∼6 Å) and very short ABTS to H451 distances (∼2 Å), whereas, for the wild type, the corresponding distances are >10 and >6 Å (Figures 4d and S11c,d), respectively. These results highlight the importance of the Met-loop in ABTS binding to cavity A and the role of cavity B in 2F4. Furthermore, they suggest that the deletion of 24 residues from the Met-loop most likely redirected the effect mutations have on protein dynamics.

### Evolutionary Trajectory Analysis Reveals Epistatic Interactions

**Figure 5:**
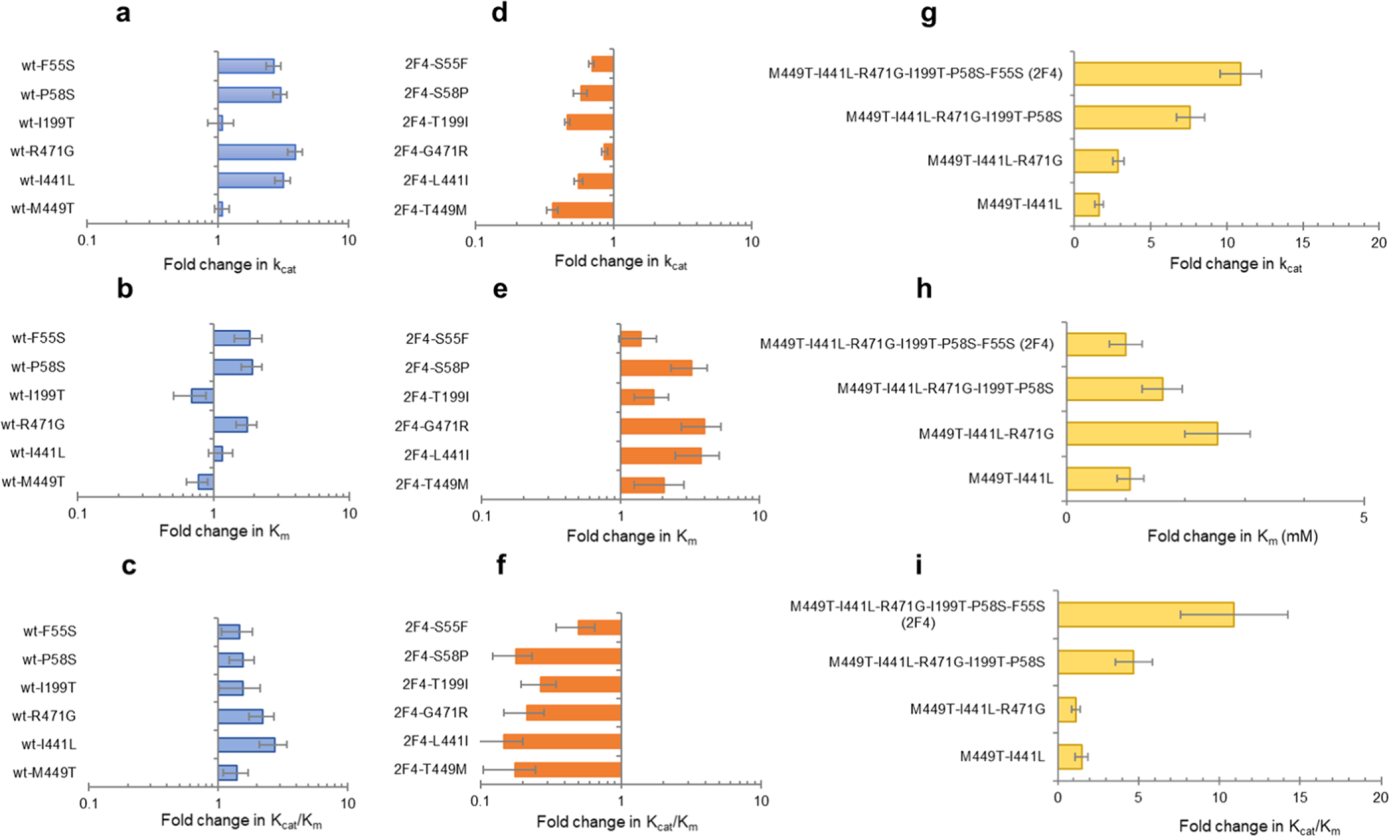
Comparison of kinetic parameters for ABTS oxidation of variants with single mutations M449T, I441L, R471G, I199T, P58S, and F55S in the wild-type background (a–c) and mutations T449M, L441I, G471R, T199I, S58P, and S55F in the 2F4 variant background (d–f), and insertion of mutations M449T, I441L, R471G, I199T, P58S, and F55S in the evolutionary trajectory (g–i).

To explore the role of the acquired mutations in shaping McoA into 2F4, we constructed single-point variants for every individual mutation obtained by DE in the wild-type background. Simultaneously, we reverted every single mutation in 2F4 back to its wild-type equivalent (Table S10). All variants in the wild-type background show similar or up to ∼4-fold higher *k*_cat_ relative to wild-type and comparable *K*_m_ values, resulting in an average 2-fold increase of the *k*_cat_/*K*_m_ (Figure 5a–c and Table S11). Variant R471G displayed the highest increase in *k*_cat_ (4-fold higher) and one of the highest *K*_m_ amongst the individual variants. The sum of effects from all mutations in the wild-type background, assuming perfect additivity, would result in a cumulative ∼20-fold increase, the double of the final ∼10-fold *k*_cat_/*K*_m_ of the 2F4 variant. This observation suggests epistasis, *i.e.*, nonadditive interactions between mutations that affect fitness.^66^ The removal of individual mutations in the 2F4 background resulted in a slight decrease of *k*_cat_ (down to ∼3-fold) and an increase of *K*_m_ values (up to 4-fold) (Figure 5d–f and Table S12), resulting in an average 6-fold decrease of the *k*_cat_/*K*_m_. Again, the R471G mutation was a case in point since its reversion in 2F4 (*i.e.*, to G471R) led to the most significant increase in the *K*_m_ value. Furthermore, insertion of the distal mutation I199T in the wild-type background increased the solubility and led to around 4-fold higher enzyme production levels (Figure S12a); its reversion in 2F4 caused a 2-fold drop in amounts of soluble protein (Figure S12b). Unlike the changes in enzyme function and solubility, the thermal stability remained unchanged in all variants (Table S13). However, the differential thermogram of variants containing mutations I199T and I441L does not reveal protein aggregation (identified by a drop in the baseline after the endothermic peak) observed in other variants (Figure S13). This shows a putative role of I199T (as well as I441L) in preventing aggregation, *e.g.*, in stabilizing the unfolded state of the enzyme.

We reconstructed the evolutionary trajectory by adding mutations in the same sequential order in which they appeared during the laboratory evolution experiments to further explore epistasis events in the course of evolution (Table S10). The kinetic analysis of variants (Figure 5g–I and Table S14) showed that inserting the R471G mutation during the second round resulted in a 2-fold increase of the *k*_cat_ but also of the *K*_m_ value similar to the effect observed upon its insertion in the wild-type background (Figure 5a–c). Notably, the introduction of the three subsequent and distant mutations, I199T, P58S in the third, and F55S in the fourth rounds, resulted in a gradual increase of the *k*_cat_ (2 and 3-fold) and a decrease of the *K*_m_ (1.5 and 2-fold), resulting in a final *k*_cat_/*K*_m_ increase of ∼5 to 10-fold as compared to the wild type. This positive influence of the distal mutations in the evolutionary pathway is significantly higher than their contributions in the context of a wild-type background, indicating the effect of long-range interactions in causing high-order epistasis.^67,68^ As observed with single variants, insertion of I199T and I441L and also of F55S mutations resulted in 2 to 3-fold higher amounts of soluble protein (Figure S12c) and may provide stability to emerging folds and promote enzyme evolvability.^69^

### Dynamical Network Analysis Highpoint Allosteric Communications

**Figure 6:**
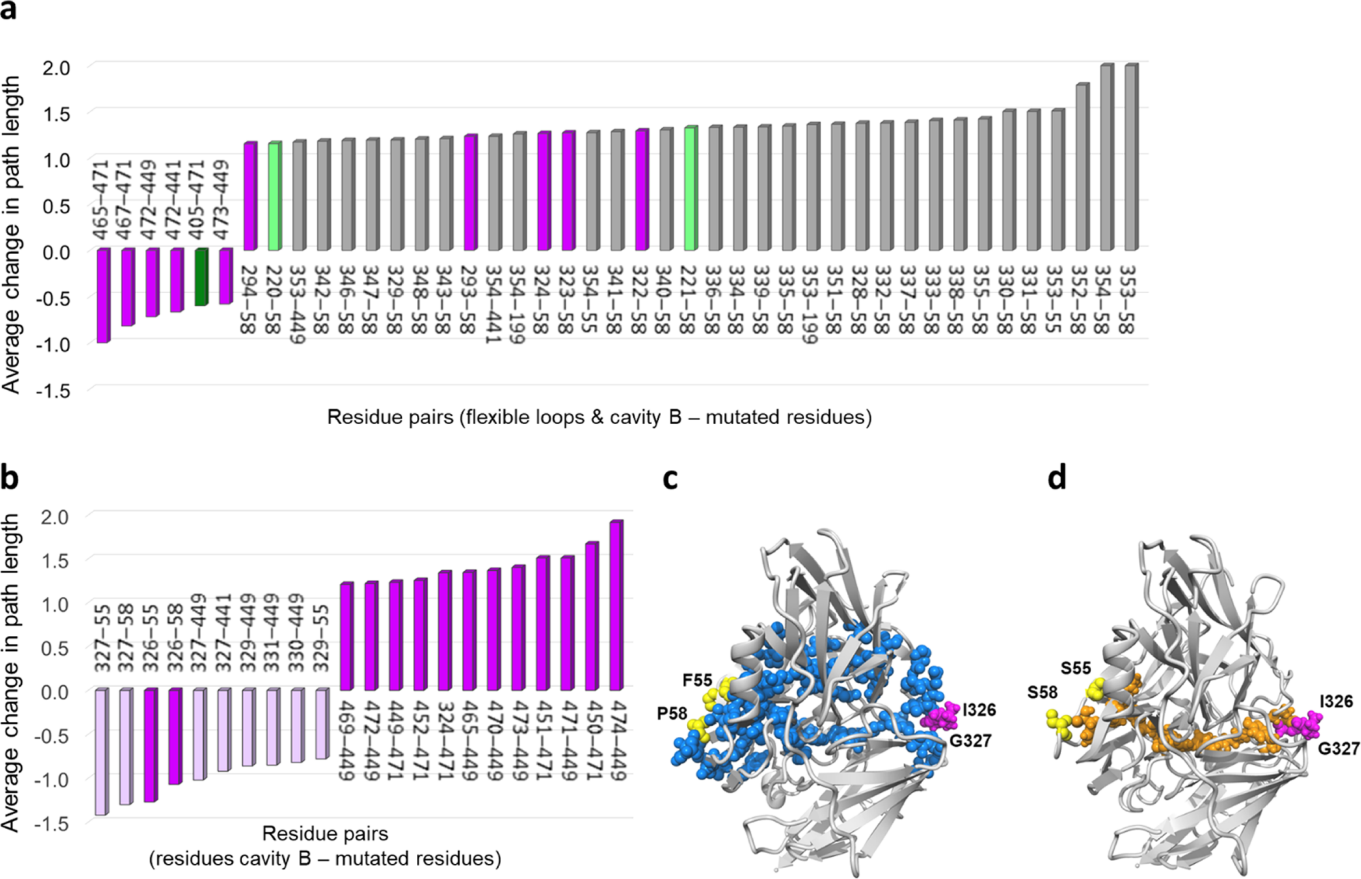
Protein residue network (PRN) analysis was performed on the MD trajectories of wild type and 2F4 (a) and of loop-truncated wild-type and 2F4 variants (b) to identify possible sources for the change in catalysis produced by distant mutations. The method was used to determine communication pathways between mutated residues and flexible loops close to cavity A and those forming the catalytically productive cavity B in 2F4. Residue–residue network optima paths that decrease and increase the most between 2F4 and wild type are given. (a) Some pathways, between mutated residues 471 and 449 and cavity B residues, which are delimiting the cavity, show a decrease in the path length, indicating an increased correlation between these residue pairs in 2F4 to wild type. However, significantly longer communication pathways (*i.e.*, less correlated dynamics, right panel) are observed upon mutation between residue 58 and most of the Met-rich loop. Interestingly, residues 353 and 354, located at the end of the loop, present significantly longer paths than other mutated residues (55, 441, and 449). (b) In loop-truncated 2F4, pathways involving residues 55 and 58 (also 449 and 441) are the ones that have decreased the most. Pathways involving 471 and 449 are the ones that show the most increase in the path length. The Met-rich loop (residues 327–355) is in gray, residues 220–226 are in light green, residues 400–407 in dark green, cavity B residues are in purple, and light purple if the residues belong both to cavity B and to the truncated five-residue loop (starting at 327), which delimits cavity B and separates it from cavity A. (c, d) Comparison between loop-truncated wild type and 2F4 lead to the identification of much shorter, direct, and focused pathways in 2F4 between residues 55 and 58 (shown in yellow spheres) and residues forming cavity B (shown in magenta). Amino acids involved in these communication pathways are represented for (c) wild type (pathways shown in blue) and (d) 2F4 (pathways shown in orange).

To single out the influence of remote mutations in shaping the function of the evolved 2F4 variant, we performed a dynamic cross-correlation (DCC) analysis of the MD simulations. The study showed that most of the changes in correlated motions between pairs of residues are located at the base and edges of the Met-loop and part of loop 220–226 near cavities A and B (Figure S14a,b). Protein residue networks (PRN) were then built connecting residues based on DCC to assess how their motions are linearly correlated. The larger their correlation, the shorter the edge connecting two residues,^63^ and optimal (shortest) and suboptimal pathways linking distinct protein residues are identified. We first analyzed how information travels from the mutated residues to the flexible loops (220–226, 401–407, and Met-loop) close to cavity A and residues in cavity B. Every residue-to-residue path length was ranked based on its variation on passing from wild type to 2F4 (Figure 6). The analysis shows that mutations M449T and R471G alter the dynamical networks of some residues that shape cavity A and particularly cavity B, promoting a more efficient ABTS binding. Interestingly, pathways that increased their length were observed in 2F4, *e.g.*, between distal mutation P58S and residues extending over the full Met-loop and loop 220–226 (Figure 6a), indicative of a loss in compelling dynamic correlation as compared to the wild-type enzyme. This suggests a distal perturbation of the communication network that has likely contributed to, *e.g.*, shifting the conformational equilibrium of the Met-loop toward a predominantly closed-conformation state in 2F4. Furthermore, PRN was performed in loop-truncated variants (loop 5) to determine communication pathways between mutated residues and those forming the catalytically productive cavity B. In the absence of the long loop, the most significant improvement in communication pathways (*i.e.*, shortened paths) is found between distant F55S and F58S mutations and residues at the basis of the Met-loop (326–331), forming cavity B (Figure 6b). The most significant increases in the path length are between mutations R471G and M449T and residues in cavity B. The results obtained expose here the complexity of the networks of interactions between residues in proteins and the mechanisms that rewire such connections. To inspect how the communication strengthened, each residue contributing to the five most shortened paths was mapped onto the protein structure of the wild-type and 2F4-truncated variants. It seems clear that most of the residues implicated in the wild type were not implicated in 2F4, while all residues involved in 2F4 are also relevant in the wild type (Figure 6c,d). The results indicate that long-range communication channels are enhanced by focusing their route in the evolved variant that most likely, *e.g.*, favored a catalytically competent conformational state of cavity B. This evolution turned a scattered and noisy communication channel into a well-defined linear pathway.

## Discussion

Biocatalysis is a vital tool for establishing future circular bioeconomies, and its application has constantly been increasing in a range of industries.^70,71^ Enzymes are specific, efficient, and green alternatives to traditional chemical catalysts. DE is a potent approach to engineer enzymes with improved properties, including catalytic efficiency and robustness toward denaturants.^72,73^ It has also been instrumental in providing mechanistic insights into protein function and evolution.^68,74−82^ In this work, the higher turnover number for ABTS of the evolved 2F4 variant was associated with alterations in substrate-binding cavities. The conformational reconfigurations close to the T1 Cu site resulted in a higher *in silico* productive ABTS binding frequency. This is foreseeable as the catalytic rate-limiting step in MCOs is substrate oxidation at the T1 Cu site. The first and second-sphere residues surrounding the T1 Cu site control both the intermolecular ET, from the substrate to the T1, and the intramolecular ET, from the T1 to the TNC.^7^ In wild-type McoA, substrates bind preferentially to cavity A where the highly flexible Met-loop interacts with the surroundings in the open- and solvent-exposed states.^16^ The optimization of the enzymatic function for ABTS in 2F4 was associated with (i) displacement of residues and short loops close to cavity A, changing the pocket access and exposing further the T1 Cu to the solvent, (ii) preferential conformation of the Met-loop over cavity A is closed states, facilitating the interaction with aromatic substrates, and (iii) emergence of a new substrate-binding site (cavity B) close to T1 Cu. Cavity B is less constrained than cavity A and resembles the binding sites found in laccases.^83^ Laccase binding sites tend to be broad and often accommodate more than one type of substrate (phenols, aryl diamines, anilines, organometallics). The mutations introduced stabilized the structural transition of the Met-loop toward closed conformers. This secondary structure element, characteristic in metallo-oxidases, emerged thus as sensitive and malleable “gate-keepers” of MCO substrate-binding sites. Furthermore, our results show that Met-loop closure facilitates interactions with the aromatic substrate without jeopardizing activity for metal ions, challenging the view that Met-loops are structural determinants of metal specificity in prokaryotic MCOs by impeding the access of larger substrates to the T1 Cu.^5,84,85^ Understanding how to enhance or make flexible sites more rigid opens new opportunities for protein engineering, applicable not only to molecular recognition but also stability and catalysis.^86−88^ Further, 2F4 increases the redox potential by approximately 11 mV compared to the wild-type McoA, which is expected to contribute to the higher activity observed with the 2F4 variant. Considering the Marcus equation, the redox potential increase observed in 2F4 could result from good binding between the substrate and the enzyme, leading to a higher electron transfer rate.^7^

The alteration of the dynamic conformational landscape emerges as an essential aspect of the functional transition of McoA in line with other studies on protein evolution.^80,89−97^ Our results align with previous findings showing that early mutations in the trajectories of laboratory evolution played a permissive role by epistatically generating or enhancing the positive role of subsequent mutations.^68,89,94^ Mutation M449T is close to both cavities A and B, and its introduction in the first round of evolution was permissive to the structural variations in residues and loops, close to the T1 Cu center in the course of evolution. The mutation R471G, in the second round of evolution, is suggested to provide a stepping stone for the enlargement of cavity B to accommodate the bulky ABTS substrate better. In the third and fourth rounds of evolution, the insertion of distal mutations I199T, P58S, and F55S paved the way for enzyme optimization through intertwined molecular epistasis, reflecting the existence of physical interaction networks. The analysis of communication pathways revealed that mutations had fine-tuned dynamic networks of residues close to the T1 Cu site during evolution, such as the dynamics of neighboring residues in cavity B and loops close to cavity A, toward states more suitable for recognizing and stabilizing ABTS. These mutations underlie protein motions and behave similarly to how small allosteric molecules introduce conformational change *via* long-range coupling.^67,80,82,91,98^ The optimization of protein function seems to require the rewiring of intramolecular networks to promote novel interactions, as observed by the differences in effective dynamic correlations of communication networks in full-length wild type and 2F4 in the respective truncated-loop variants. This spots the importance of iterative experimental and computational analyses in providing mechanistic insight into local-to-global changes involved in the evolution of enzyme specificity.

In conclusion, the molecular basis of substrate specificity and the dynamics and constraints of the evolution of a hyperthermophilic metallo-oxidase McoA to “unnatural” aromatic substrates were unveiled in this work. A combination of mutagenesis, kinetics, X-ray diffraction, SAXS, and molecular modeling allows mapping the sequence–structure–function relations, identifying epistatic interactions, and deciphering its biophysical basis, such as the residue-to-residue communication networks that underlie allosteric and dynamic fine-tuning of substrate-binding sites for bulkier substrates. This undertaking contributed to advancing our knowledge of the features and mechanisms behind the different substrate specificities in MCOs, understanding evolutionary dynamics, and fostering the design of new proteins.

## Supporting Information Available

Primer information (Table S1); X-ray data collection, processing, and refinement statistics (Table S2); SEC-SAXS data collection and analysis (Table S3); protein production, copper content, and molar coefficients at 600 nm for wild-type McoA and variants (Tables S4 and S10); kinetic parameters for ABTS, Cu(I), and Fe(II) of wild-type and evolved variants (Table S5); sequence analysis, activity, and stability of the most active variants obtained after DNA shuffling (Table S6); cavities A and B close to T1 Cu in the wild-type and 2F4 variant (Table S7); solvent accessible surface area (ASA) of residues in cavity A and B (Table S8); steady-state kinetic parameters for ABTS of the wild-type and loop-truncated variants (Table S9); steady-state kinetic parameters for ABTS of purified single variants M44T, I441L, I199T, P58S, and F55S (Table S11); steady-state kinetic parameters for ABTS of variants constructed eliminating each single mutation from the 2F4 variant (Table S12); melting temperatures (*T*_m_) of McoA and variants by differential scanning calorimetry (DSC) (Table S13); steady-state kinetic parameters for ABTS of variants constructed during the reconstruction of the evolution trajectory (Table S14); figures showing the spectroscopic, kinetic, and stability feature comparison among wild type and variants 2B3, 2B3wsp, and 2F4 (Figure S1); structural comparison of catalytic centers of wild type and 2F4 (Figure S2); ABTS binding sites in prokaryotic MCOs (Figure S3); SEC-SAXS profile for 2F4 (Figure S4); Met-loop modeling (Figure S5); Met-loop probed by molecular dynamic simulations (Figure S6); cavity size and accessibility from the simulations (Figure S7); molecular representation of ABTS binding in the proximity of the T1 Cu for wild type and 2F4 (Figure S8); local dockings of ABTS to wild type and 2F4 (Figure S9); docking of ABTS to cavity A and cavity B of Met-loop-truncated variants (Figure S10); local docking of ABTS to cavity A and B of the loop-truncated variants (Figure S11); soluble protein fractions and production yields of variants (Figure S12); excess heat capacity of wild type and variants (Figure S13); and dynamical cross-correlation (DCC) maps (Figure S14) (PDF)
