# Supplementary material for: Distal Mutations Shape Substrate-Binding Sites during Evolution of a Metallo-Oxidase into a Laccase
Source: ACS Catal. 2022 Apr 13;12(9):5022–35. doi: 10.1021/acscatal.2c00336 (PMC9775220; doi:10.1021/acscatal.2c00336)
Supplement: Supplementary file 1 — cs2c00336_si_001.pdf [file cs2c00336_si_001.pdf]

SUPPORTING INFORMATION

Distal Mutations Shape Substrate-Binding Sites during Evolution of a Metallo-Oxidase into a Laccase

Vânia Brissos[1†], Patrícia T. Borges[1†], Reyes Núñez-Franco [2], Maria Fátima Lucas[2], Carlos Frazão[1], Emanuele Monza[2], Laura Masgrau[2,3], Tiago N. Cordeiro[1], Lígia O. Martins[1]*

[1]*Instituto de Tecnologia Química e Biológica António Xavier, Universidade Nova de Lisboa, Av da República, 2780-157 Oeiras Portugal*

[2]*Zymvol Biomodeling, Carrer Roc Boronat, 117, 08018 Barcelona, Spain*

[3]*Department of Chemistry, Universitat Autònoma de Barcelona, 08193 Bellaterra, Spain*

† These authors contributed equally to this work

***Corresponding author:** lmartins@itqb.unl.pt

Table S1. Summary of primers used for the construction of 2B3 without the signal peptide, loop-truncated variants, and variants constructed by using site-directed mutagenesis. F indicates forward primers and R indicates reverse primers.

Name	Sequence	Reference
mcoA-T43M	5'-CATATGTTGAGTAAACAATCCCTCAATATCCCC-3'	[1]
mcoA-1816R	5'-GACTTAGAATTCTCAACATATTGCACC-3'	[2]
pET21D	5’-CTTCCCCATCGGTGATGTCGGCGATATAG-3’	This work
pET21R	5’-CCAAGGGGTTATGCTAGTTATTGCTCAG-3’	This work
M449T F	5'-CGTAAACAACACGGGTACCTACCACCCCATGCACATAC-3'	[3]
M449T R	5'-GTATGTGCATGGGGTGGTAGGTACCCGTGTTGTTTACG-3'	[3]
I441L F	5'-GGCGACGTGGTGATTTAGAGTACGTAAACAACACG-3'	[3]
I441L R	5'-CGTGTTGTTTACGTACTCTTAAATCACCACGTCGCC-3'	[3]
R471G F	5'-GAAAGGAGCTTGGGACCTTTGGGGGCTACGGACCTCGG-3'	[3]
R471G R	5'-CCGAGGTCCGTAGCCCCCAAAGGTCCCAAGCTCCTTTC-3'	[3]
I199T F	5'-CTCGAATACGGAGTTACAGACATTCCGCTCATAATTCAGG-3'	[3]
I199T R	5'-CCTGAATTATGAGCGGAATGTCTGTAACTCCGTATTCGAG-3'	[3]
P58S F	5'-CGGATACTTCCTTTTTTCCCGATGGACAGCGAGTAAG-3'	[3]
P58S R	5'-CTTACTCGCTGTCCATCGGAAAAAAGGAAGTATCCG-3'	[3]
F55S F	5'-CAATATCCCCGGATACTCCCTTTTTCCCGATGGAC-3'	[3]
F55S R	5'-GTCCATCGGGAAAAAGGGAGTATCCGGGGATATTG-3'	[3]
F55SP58S F	5'-CAATATCCCCGGATACTCCCTTTTTTCCGATGGACAGCGAGTAAG-3'	[3]
F55SP58S R	5'-CTTACTCGCTGTCCATCGGAAAAAAGGGAGTATCCGGGGATATTG-3'	[3]

T449M F	5'-CGTAAACAACACGGGTATGTACCACCCCATGCACATAC-3'	[3]
T449M R	5'-GTATGTGCATGGGGTGGTACATACCCGTGTTGTTTACG-3'	This work
L441I F	5'-GGCGACGTGGTGATTATAGAGTACGTAAACAACACG-3'	This work
L441I R	5'-CGTGTTGTTTACGTACTCTATAATCACCACGTCGCC-3'	This work
G471R F	5'-GAAAGGAGCTTGGGACCTTTGAGGGCTACGGACCTCGG-3'	This work
G471R R	5'-CCGAGGTCCGTAGCCCTCAAAGGTCCCAAGCTCCTTTC-3'	This work
T199I F	5'-CTCGAATACGGAGTTATAGACATTCCGCTCATAATTCAGG-3'	This work
T199I R	5'-CCTGAATTATGAGCGGAATGTCTATAACTCCGTATTCGAG-3'	This work
S58P F	5'-CAATATCCCCGGATACAGCCTTTTTCCCGATGGACAGCGAGTAAG-3'	This work
S58P R	5'-CTTACTCGCTGTCCATCGGGAAAAAGGCTGTATCCGGGGATATTG-3'	This work
S55F F	5'-CAATATCCCCGGATACTTCCTTTTTAGCGATGGACAGCGAGTAAG-3'	This work
S55F R	5'-CTTACTCGCTGTCCATCGCTAAAAAGGAAGTATCCGGGGATATTG-3'	This work
mcoA Δ336-345 (loop 19) R	5’-CATTCTCATTCCAATCATTCCCATTCC-3'	[1]
mcoA Δ336-345 (loop 19) F	5’-GGAAATGGAATGAATATGGATATGGGTATGG-3'	[1]
mcoA Δ333-348 (loop 13) R	5’-TCCAATCATTCCCATTCCTATTAAGTTG-3'	[1]
mcoA Δ333-348 (loop 13) F	5’-ATGAATATGGATATGGGTATGGCAGATAAC-3'	[1]
mcoA Δ330-351 (loop 7) R	5’-TCCCATTCCTATTAAGTTGTGAGGGAAG-3'	[1]
mcoA Δ330-351 (loop 7) F	5’-GATATGGGTATGGCAGATAACTCAGAGTTTG-3'	[1]
mcoA Δ329-352 (loop 5) R	5’-CATTCCTATTAAGTTGTGAGGGAAGTTG-3'	[1]
mcoA Δ329-352 (loop 5) F	5’-ATGGGTATGGCAGATAACTCAGAGTTTG-3'	[1]

Table S2. X-ray data collection, processing and refinement statistics of 2F4 variant. Values in parentheses belong to the highest resolution shell.

**Data Collection**	
Beamline	ID23-1
Wavelength (Å)	0.9762
Space group	*P2*
Unit cell parameters (Å, °)	a = 63.2, b = 89.6, c = 86.9 ; β = 106.1
Resolution (Å)	49.41-1.80 (1.90-1.80)
Number of observations	255823 (22156)
Unique reflections	78045 (8715)
Completeness (%)	99.0 (97.5)
Multiplicity	3.3 (2.5)
Mosaicity (°)	0.11
$CC_{1/2}$ (%)[a]	98.7 (39.4)
R_{sym} (%)[b]	9.2 (49.7)
R_{meas} (%)[c]	12.5 (103.6)
R_{pim} (%)[d]	7.4 (34.4)
$\langle I/\sigma(I)\rangle$	7.21 (1.31)
Wilson B-factor (Å^2)	18.0
V_M (Å^3 Da^{-1})	1.99
Estimated solvent content (%)	38.3
**Refinement**	
R_{factor} (%)[e]	16.0
R_{work} (%)[f]	16.0
R_{free} (%)[f]	19.8
rmsd for bond lengths (Å)	0.015
rmsd for bond angles (°)	1.370
Structure <*a.d.p.*> (Å^2)	20.2
O_2 <*a.d.p.*> (Å^2)	13.5
Cu1, Cu2, Cu3 and Cu4 <*a.d.p.*> (Å^2)	14.7, 15.3, 14.6, 16.8
Number of residues	457
Number of solvent waters	792
Ramachandran plot	
Residues in favored regions (%)	97.5
Residues in allowed regions (%)	2.5
Residues in disallowed regions (%)	0
PDB code	6TTD

[a] $CC_{1/2}$ = Percentage of correlation between intensities from random half-datasets [4]

[b] $R_{sym} = \Sigma_{hkl} \Sigma_i |I_i(hkl) - \langle I(hkl)\rangle| / \Sigma_{hkl} \Sigma_i I_i(hkl)$, where $I_i(hkl)$ is the observed intensity and $\langle I(hkl)\rangle$ is the average intensity of multiple observations from symmetry-related reflections. [5]

[c] $R_{meas} = \Sigma_{hkl} [N/(N(hkl) -1)]^{1/2} \Sigma_i |I_i(hkl) - \langle I(hkl) \rangle| / \Sigma_{hkl} \Sigma_i I_i (hkl)$, where N(hkl) is the data multiplicity, $I_i(hkl)$ is the observed intensity and $\langle I(hkl) \rangle$ is the average intensity of multiple observations from symmetry-related reflections. It is an indicator of the agreement between symmetry related observations. [6]

[d] $R_{p.i.m.} = \Sigma_{hkl} [1/(N(hkl) -1)]^{1/2} \Sigma_i |I_i(hkl) - \langle I(hkl) \rangle| / \Sigma_{hkl} \Sigma_i I_i (hkl)$, where N(hkl) is the data multiplicity, $I_i(hkl)$ is the observed intensity and $\langle I(hkl) \rangle$ is the average intensity of multiple observations from symmetry-related reflections. It is an indicator of the precision of the final merged and averaged data set. [7]

[e] $R_{factor} = \Sigma |F_{obs} - F_{calc}| / \Sigma F_{obs}$, where F_{obs} and F_{calc} are the amplitudes of the observed and the model calculated structure factors, respectively. It is a measure of the agreement between the experimental X-ray diffraction data and the crystallographic model.

[f] R_{work} refers to the actual working data set used in refinement, while R_{free} refers to a cross validation set that is not directly used in refinement and is therefore free from refinement bias.

Table S3. SEC-SAXS data collection and analysis.

	**2F4**	**2F4-Loop5** (2F4Δ329-352)
**Data acquisition**		
Beamline – Facility	BM29-ESRF	BM29-ESRF
Wavelength (Å)	0.99	0.99
Sample-to-detector distance (m)	2.87	2.87
s range ($Å^{-1}$)	0.0038-0.4920	0.0038-0.4920
Concentration ($mg{\cdot}mL^{-1}$)	~10.0	~10.0
HPLC system / SEC column	Shimadzu / Superdex 200 10/300	Shimadzu / Superdex 200 10/300
Detector	Pilatus 1M	Pilatus 1M
Temperature (K)	293.15	293.15
**Overall parameters**		
R_g (Å) [from *P(r)*]	22.63 ± 0.01	22.02± 0.01
R_g (Å) [from Guinier]	22.61 ± 0.04	22.10± 0.12
D_{max} (Å)	68.35 ± 0.35	66.31 ± 0.20
Porod volume estimate, *Vp* ($Å^3$)	78129.6	74318.4
Molecular weight estimate (kDa)[a]	49.1 (11.1%)	45.0 (14.6%)
Oligomeric state	Monomer	Monomer
**Software**		
SEC-SAXS data integration	ScÅtter	ScÅtter
P(r)	GNOM 5.0	GNOM 5.0
Ab initio Modelling / <NSD> (Å)[b]	DAMMIF[c] / 0.53 ± 0.19 Å	DAMMIF[c] / 0.56 ± 0.20 Å
Simulated SAXS	CRYSOL	CRYSOL
**SASBDB accession code**	SASDHL8	SASDHM8

[a] Calculated with SAXSMoW 2.1 [8]. The discrepancy to the sequence weight is given inside parentheses.
[b] Mean ± STD
[c] Refinement with DAMMIF [9]

Table S4. Protein production, copper content, molar coefficients at 600 nm and redox potential for wild-type (WT) without signal peptide and variants 2B3 with and without signal peptide (23wsp), and the hit variant 2F4 obtained after DNA shuffling.

variants	Protein Production (mg/L)	Copper content (mol of Cu/mol of protein)	$\varepsilon_{600\ nm}$ ($mM^{-1}\ cm^{-1}$)	Redox Potential (mV)
WT	0.7 ± 0.3	4.0 ± 0.4	3.7	534
2B3	2.5 ± 0.5	4.5 ± 0.5	3.3	-
2B3 wsp	2.9 ± 0.6	3.8 ± 0.4	3.7	-
2F4	4.9 ± 1.1	4.0 ± 0.1	3.7	545

Table S5. Apparent steady-state kinetic parameters for ABTS, and metal ions Cu(I) and Fe(II) of the purified wild-type (WT) without signal peptide and variants 2B3 with and without signal peptide, and the hit variant 2F4 obtained after DNA shuffling. Reactions were performed at 40ºC. The results are reported as mean ± standard error for k_{cat} and K_m. Errors for k_{cat}/K_m were obtained by propagation.

	ABTS			Cu(I)			Fe(II)		
variants	k_{cat} (s^{-1})	K_m (mM)	k_{cat}/K_m ($s^{-1}\cdot M^{-1}$)	k_{cat} (s^{-1})	K_m (mM)	k_{cat}/K_m ($s^{-1}\cdot M^{-1}$)	k_{cat} (s^{-1})	K_m (mM)	k_{cat}/K_m ($s^{-1}\cdot M^{-1}$)
WT	25 ± 3	1.3 ± 0.2	$(1.9 \pm 0.5) \times 10^4$	34 ± 7	0.030 ± 0.005	$(11 \pm 1) \times 10^5$	35 ± 8	0.012 ± 0.002	$(2.9 \pm 0.9) \times 10^6$
2B3	178 ± 16	1.4 ± 0.3	$(1.3 \pm 0.3) \times 10^5$	nd	nd	nd	nd	nd	nd
2B3wsp	192 ± 27	0.9 ± 0.1	$(2.1 \pm 0.4) \times 10^5$	nd	nd	nd	nd	nd	nd
2F4	273 ± 9	1.5 ± 0.4	$(1.8 \pm 0.5) \times 10^5$	22 ± 6	0.026 ± 0.005	$(8.5 \pm 0.3) \times 10^5$	35 ± 5	0.022 ± 0.007	$(1.6 \pm 0.6) \times 10^6$

nd- not determined

Table S6. Sequence analysis, activity for ABTS at 40°C and half-life at 90°C, of the nine most active variants obtained after DNA-shuffling between the gene coding for 2B3 and wild-type. The activity and stability assays were performed in partially purified protein preparations, after heating crude extracts at 80ºC. Synonymous mutations are presented in parentheses.

**Variants**	**2B3wsp**	**2F4**	**6D9**	**1B10**	**8F6**	**7C4**	**8E4**	**5B6**	**6C8**	**2D6**
V_{max} ($U\cdot mg^{-1}$)	1.4 ± 0.1	2.1 ± 0.1	0.66 ± 0.04	0.47 ± 0.01	0.42 ± 0.03	0.39 ± 0.02	0.21 ± 0.01	0.1 ± 0.04	0.1 ± 0.03	0.1 ± 0.01
$t_{1/2}$ 90°C (h)	6.1 ± 0.9	5.9 ± 0.9	6.9 ± 0.9	5.8	6.4 ± 0.4	6.1 ± 1	4.2 ± 0.7	5.8 ± 0.7	7.2	6.1
M449T	×	×	×	×		×	×			
I441L	×	×	×	×						
K246R	×		×	×	×		×	×		×
R471G	×	×		×					×	
I199T	×	×	×		×	×	×	×		×
Y172C	×		×	×	×	×	×	×	×	×
P58S	×	×		×	×	×	×		×	×
F55S	×	×			×	×	×		×	×
(G255G)	×		×	×	×	×	×	×		×
(Q380Q)	×		×		×		×		×	×
(G38G)	×									
(E412E)	×	×			×					×
(V444V)	×	×	×	×		×	×			

Table S7. Molecular dimensions and amino acid residues that limit cavities A and B close to T1 Cu center in wild-type and 2F4 structures.

		Area (Å^2)	Volume (Å^3)	Depth (Å)	Residues
**Cavity A**	Wild-type	388.41	194.52	9.37	G164, R165, T166, G167, Y168, Y218, P220, M221, G222, H223, M224, G225, F226, V406, F407, E513, **H514**, D516, E517
	2F4	354.75	179.97	13.2	G164, R165, G167, Y168,Y218, P220, M221, G222, H223, M224, G225, F226, F407, I511, E513, **H514**, D516, E517
**Cavity B**	Wild-type	221.16	122.05	7.26	R262, L294, N324, L325, R465, L467, G468, P469, R471, I482, A484, E487
	2F4	360.94	436.23	13.01	R262, V276, V290, E292, I293, L294, E299, I301, L325, R465, L467, G471, A472, T473, D474, K478, T480, V481, I482, A484, E487

Table S8. Solvent accessible surface areas (ASA) of residues part of cavities A and B close to T1 Cu site in wild-type and 2F4 structures. NA means that is not applicable.

**Cavity A**			**Cavity B**		
	ASA (%)			ASA (%)	
Residues	Wild-type	2F4	Residues	Wild-type	2F4
G164	29	28	R262	19	19
R165	39	39	V276	NA	1
T166	1	NA	V290	NA	16
G167	1	1	E292	NA	32
Y168	19	21	I293	NA	1
Y218	23	21	L294	3	17
P220	33	39	E299	NA	1
M221	47	69	I301	NA	1
G222	59	22	N324	61	NA
H223	29	14	L325	7	5
M224	39	22	R465	5	14
G225	1	2	L467	56	62
F226	1	1	G468	43	NA
V406	32	NA	P469	84	NA
F407	8	3	R/G471	32	46
I511	NA	1	A472	NA	15
E513	5	6	T473	NA	12
H514	0.2	1	D474	NA	9
D516	14	18	K478	NA	1
E517	23	34	T480	NA	1
			V481	NA	1
			I482	2	5
			A484	11	14
			E487	2	3

Table S9. Steady-state kinetic parameters for ABTS of loop-truncated variants of wild-type (WT) and 2F4. Reactions were performed at 40ºC. The results are reported as mean ± standard error for k_{cat} and K_m. Errors for k_{cat}/K_m were obtained by propagation.

variants	k_{cat} (s^{-1})	K_m (mM)	k_{cat}/K_m ($s^{-1}\cdot M^{-1}$)
WT	25 ± 3	1.3 ± 0.2	(1.9 ± 0.4) × 10^4
WT-loop 19	114 ± 5	2.0 ± 0.4	(5.7 ± 1.2) × 10^4
WT-loop 13	114 ± 2	3.5 ± 0.2	(3.3 ± 0.2) × 10^4
WT-loop 7	94 ± 4	4.2 ± 0.6	(2.2 ± 0.3) × 10^4
WT-loop 5	110 ± 2	3.6 ± 0.2	(3.1 ± 0.2) × 10^4
2F4	273 ± 9	1.5 ± 0.4	(1.8 ± 0.5) × 10^5
2F4-loop 19	113 ± 3	2.0 ± 0.2	(5.7 ± 0.6) × 10^4
2F4-loop 13	172 ± 4	2.5 ± 0.3	(6.9 ± 0.8) × 10^4
2F4-loop 7	83 ± 13	4.0 ± 0.5	(2.1 ± 0.4) × 10^4
2F4-loop 5	133 ± 20	3.8 ± 0.6	(3.5 ± 0.8) × 10^4

Table S10. Copper content, molar coefficients at 600 nm and protein production for wild-type McoA (WT) and variants studied in this work.

variants	Copper content (mol of Cu/mol of protein)	$\varepsilon_{600\ nm}$ ($mM^{-1}\ cm^{-1}$)	Protein production ($mg\ L^{-1}$)
WT	4.0 ± 0.4	3.7	0.7 ± 0.3
M449T	4.4 ± 0.8	2.5	0.6 ± 0.2
I441L	4.6 ± 0.1	2.7	0.3 ± 0.2
R471G	3.9 ± 0.2	4.5	0.2 ± 0.1
I199T	4.6 ± 0.3	2.9	2.3 ± 0.5
P58S	4.6 ± 0.4	3.0	0.4 ± 0.2
F55S	4.6 ± 0.1	3.0	0.7 ± 0.3
M449T-I441L	4.1 ± 0.1	2.9	0.6 ± 0.3
M449T-I441L-R471G	4.1 ± 0.5	4.6	0.5 ± 0.2
M449T-I441L-R471G-I199T-P58S	4.0 ± 0.1	3.4	3.4 ± 0.9
M449T-I441L-R471G-I199T-P58S-F55S (**2F4**)	4.0 ± 0.1	3.7	4.9 ± 1.1
I441L-R471G-I199T-P58S-F55S (2F4-M449)	4.6 ± 0.6	4.3	2.0 ± 0.3
M449T-R471G-I199T-P58S-F55S (2F4-I441)	4.0 ± 0.5	4.0	4.0 ± 1.5
M449T-I441L-I199T-P58S-F55S (2F4-R471)	4.6 ± 0.2	3.8	2.6 ± 0.8
M449T-I441L-R471G-P58S-F55S (2F4-I199)	4.6 ± 0.3	3.8	1.7 ± 0.3
M449T-I441L-R471G-I199T-F55S (2F4-P58)	4.6 ± 0.4	3.7	5.4 ± 0.6
M449T-I441L-R471G-I199T-P58S (2F4-F55)	4.0 ± 0.1	3.4	2.9 ± 0.5
2F4-loop19	4.0 ± 0.1	3.7	4.2 ± 1.0
2F4-loop13	3.6 ± 0.2	6.5	7.1 ± 1.5
2F4-loop7	4.6 ± 0.3	4.2	6.8 ± 1.0
2F4-loop5	3.7 ± 0.5	4.2	3.2 ± 0.9

Table S11. Steady-state kinetic parameters for ABTS of the purified wild-type (WT) and variants M44T, I441L, I199T, P58S and F55S. Reactions were performed at 40ºC. The results are reported as mean ± standard error for k_{cat} and K_m. Errors for k_{cat}/K_m were obtained by propagation.

variants	k_{cat} (s^{-1})	K_m (mM)	k_{cat}/K_m ($s^{-1}\cdot M^{-1}$)
WT	25 ± 3	1.3 ± 0.2	(1.9 ± 0.5) × 10^4
WT-M449T	27 ± 1	1.0 ± 0.1	(2.7 ± 0.3) × 10^4
WT-I441L	79 ± 5	1.5 ± 0.2	(5.3 ± 0.8) × 10^4
WT-R471G	98 ± 4	2.3 ± 0.2	(4.3 ± 0.4) × 10^4
WT-I199T	27 ± 5	0.9 ± 0.2	(3.0 ± 0.9) × 10^4
WT-P58S	75 ± 3	2.5 ± 0.2	(3.0 ± 0.3) × 10^4
WT-F55S	67 ± 4	2.4 ± 0.4	(2.8 ± 0.5) × 10^4

Table S12. Steady-state kinetic parameters for ABTS of the purified variants constructed eliminating each single mutation from the 2F4 variant. Reactions were performed at 40ºC. The results are reported as mean ± standard error for k_{cat} and K_m. Errors for k_{cat}/K_m were obtained by propagation.

variants	k_{cat} (s^{-1})	K_m (mM)	k_{cat}/K_m ($s^{-1}\cdot M^{-1}$)
2F4	273 ± 9	1.5 ± 0.4	(1.8 ± 0.5) × 10^5
2F4-T449M	99 ± 8	3.1 ± 0.9	(3.2 ± 0.9) × 10^4
2F4-L441I	152 ± 10	5.7 ± 1.3	(2.7 ± 0.6) × 10^4
2F4-G471R	235 ± 9	6 ± 1	(3.9 ± 0.7) × 10^4
2F4-T199I	127 ± 3	2.6 ± 0.2	(4.9 ± 0.4) × 10^4
2F4-S58P	158 ± 18	4.9 ± 0.6	(3.2 ± 0.5) × 10^4
2F4-S55F	190 ± 5	2.1 ± 0.3	(9 ± 1) × 10^4

Table S13. Melting temperatures (Tm) of McoA wild-type (WT) and variants by differential scanning calorimetry (DSC). T_m values were calculated using the first derivative of enthalpy (Cp).

variants	Tm (°C)
WT	90 ± 1
WT-M449T	90 ± 2
WT-I441L	90 ± 2
WT-R471G	90 ± 2
WT-I199T	92 ± 1
WT-P58S	92 ± 1
WT-F55S	92 ± 1
2F4	93 ± 1
2F4-T449M	93 ± 1
2F4-L441I	93 ± 1
2F4-G471R	93 ± 1
2F4-T199I	93 ± 1
2F4-S58P	93 ± 1
2F4-S55F	93 ± 1
WT-M449T-I441L	93 ± 1
WT-M449T-I441L-R471G	93 ± 1
WT-M449T-I441L-R471G-I199T	93 ± 1
WT-M449T-I441L-R471G-I199T-P58S	93 ± 1
WT-M449T-I441L-R471G-I199T-P58S-F55S (2F4)	93 ± 1

Table S14. Steady-state kinetic parameters for ABTS of the purified wild-type and variants constructed during the reconstruction of the 2F4 variant. Reactions were performed at 40ºC. The results are reported as mean ± standard error for k_{cat} and K_m. Errors for k_{cat}/K_m were obtained by propagation.

enzymes	k_{cat} (s^{-1})	K_m (mM)	k_{cat}/K_m ($s^{-1}\cdot M^{-1}$)
Wild-type	25 ± 3	1.3 ± 0.2	(1.9 ± 0.5) × 10^4
M449T-I441L	40 ± 5	1.4 ± 0.2	(2.7 ± 0.3) × 10^4
M449T-I441L-R471G	83 ± 10	3.3 ± 0.5	(2.5 ± 0.5) × 10^4
M449T-I441L-R471G-I199T-P58S	190 ± 5	2.1 ± 0.3	(9 ± 1) × 10^4
M449T-I441L-R471G-I199T-P58S-F55S (2F4)	273 ± 9	1.5 ± 0.4	(1.8 ± 0.5) × 10^5

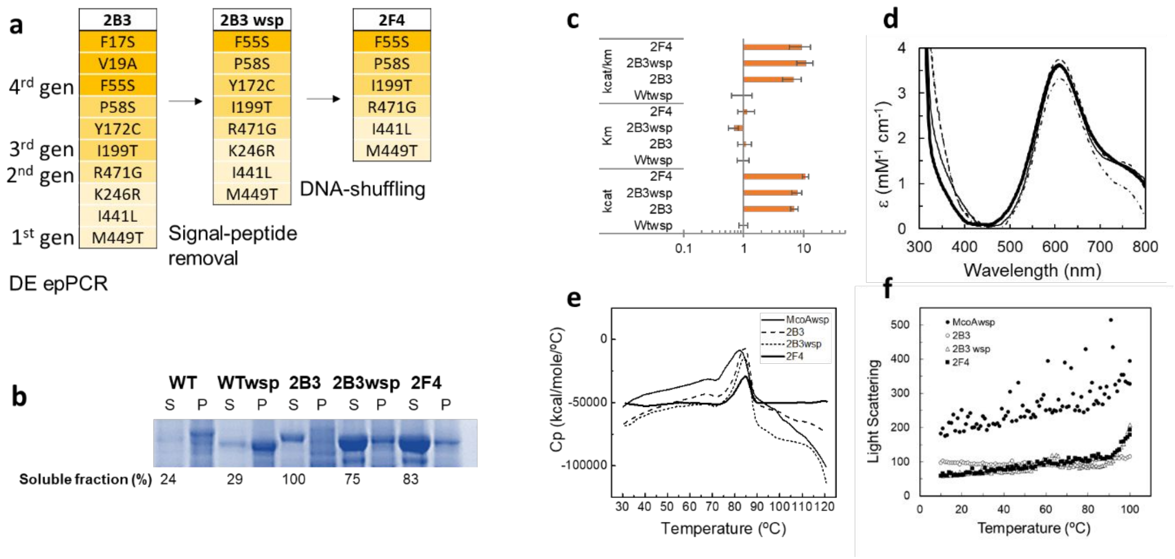

Figure S1. (a) Illustration of mutations present in the 2B3 variant obtained after four rounds of DE3, in the 2B3wsp variant, after removal of the signal peptide, and in 2F4 variant, obtained after DNA shuffling of 2B3wsp and wild-type. **(b)** SDS-PAGE gel analysis of the soluble (S) and insoluble (P) fractions of partially purified crude extracts (after heating at 80ºC) of wild type (with and without signal peptide (WT and WTwsp)), 2B3 variant (with and without signal peptide (2B3 and 2B3wsp)) and 2F4 variant. The percentage of enzyme in the soluble fraction was determined by the relative intensities of bands. Wild-type and 2B3 enzymes migrate as bands of ~ 59 kDa while WTwsp, 2B3wsp and 2F4 as bands of ~ 55 kDa, close to the theoretical values predicted from the mcoA gene sequence with (59.5 kDa) or without (55.5 kDa) the signal peptide. **(c)** Fold change of the kinetic parameters of wild type and variants determined for ABTS. **(d)** UV-visible of wild type without signal peptide (solid thin line) and variants 2B3 (dashed line), 2B3wsp (dotted line) and 2F4 (solid thick line). **(e)** Excess heat capacity obtained from DSC for wild type (thin line) and variants 2B3 (dashed line), 2B3wsp (dotted line) and 2F4 (thick solid line). The melting temperature (Tm) of wild type is 90°C while variants 2B3 and 2F4 exhibited a Tm of 93°C. **(f)** Static light scattering of wild type (closed circles) and variants 2B3 (open circles), 2B3wsp (open triangles) and 2F4 (closed squares) showing a higher propensity of wild type for aggregation.

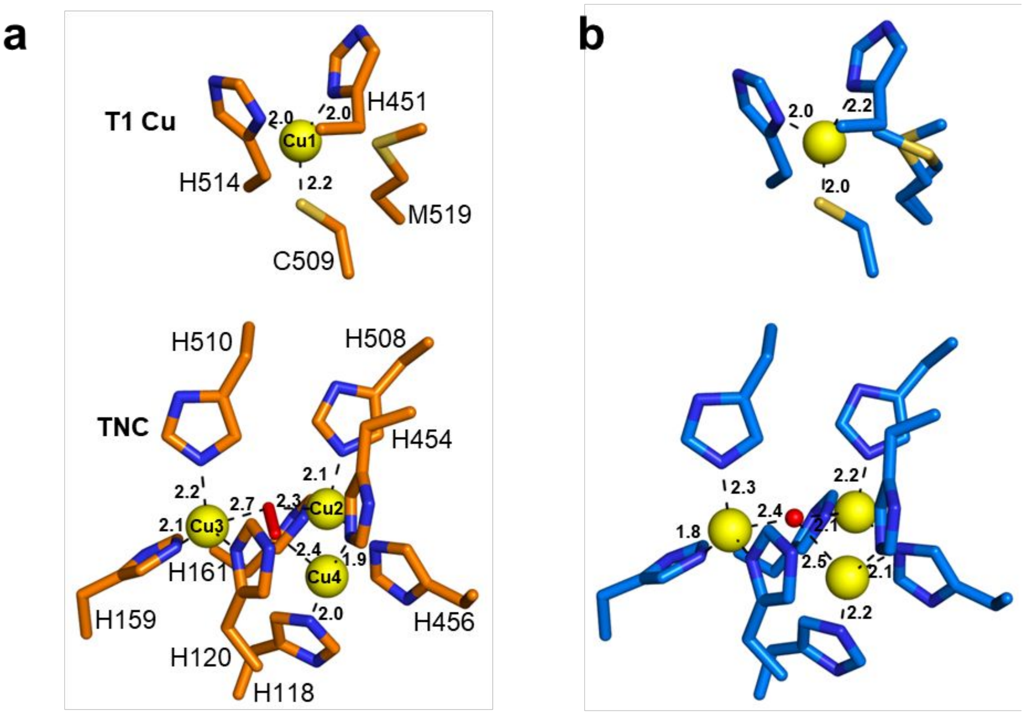

Figure S2. Catalytic centers of wild-type and 2F4. T1 Cu and TNC site from **(a)** 2F4 and **(b)** wild-type. In contrast to 2F4, the T1 Cu site ligand M519 of wild-type shows two-fold disorder at the dihedral side-chain angle χ^3 that alternates between Gauche (-) and Trans conformations with occupancies of 0.41 and 0.59, respectively. The two conformers stay at 3.8 and 3.2 Å distances of the T1 Cu site, respectively. In 2F4, the M519 side-chain assumes only the Trans conformation and residues at 3.3 Å of the T1 Cu site, and thus presents a slightly different distribution of distances to the neighboring atoms. Both crystal structures show additional electron densities at a homologous pocket near the TNC, however, while the extra electron density of 2F4 fits a di-oxygen molecule that of wild-type corresponded to a water molecule. Structures refinement led to *a.d.p.*s. values comparable with their neighboring molecules and Cu centers (Supplementary Table 4). The limited 1.8 Å resolution does not allow to distinguish among possible oxidation states of di-oxygen species, di-oxygen, peroxide or superoxide, nevertheless refinements trials with the two last species led to uneven difference Fourier maps, while that of di-oxygen produced smooth maps. The di-oxygen molecule found in 2F4 corresponds to an unreacted substrate in a crystal produced at pH 5.5, which activity is reduced to 15%. The water molecule found in wild-type crystal was observed within the enzyme optimal pH 4.0-4.5. 2F4 has two independent molecules and the respective pockets where di-oxygen is located are not exactly the same which leads to a different orientation of this molecule. The copper atoms are represented as bright yellow spheres with their bond distances (Å) in black dashed lines and their ligands as sticks with carbon, nitrogen, oxygen and sulfur atoms colored in orange, dark blue, red and yellow, respectively. Wild-type has a similar representation but with carbon sticks in blue. The water and di-oxygen molecules are represented as a red sphere and stick, respectively.

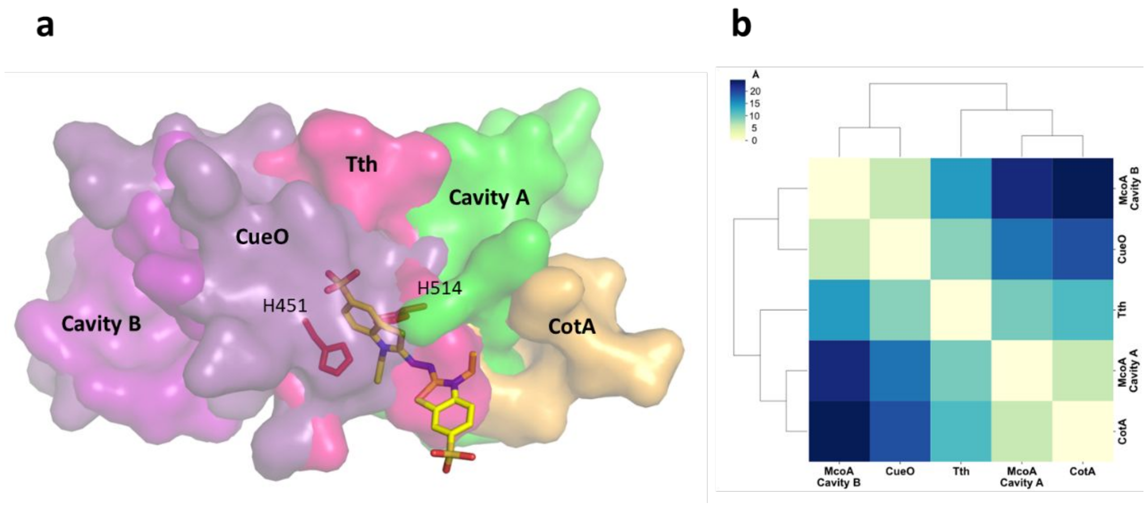

Figure S3. ABTS binding sites in prokaryotic MCOs. **(a)** Cavities are shown as ASA. Cavities A and B from 2F4 are colored in green and purple, respectively. The T1Cu ligands H451 and H514 are shown as red sticks. The binding site for ABTS in *B. subtilis* CotA (PDB 3ZDW) is shown in orange. The ABTS molecule identified in this structure is shown as sticks with carbon, nitrogen and oxygen in yellow, blue and red, respectively. The predicted binding site for ABTS in *E. coli* CueO (PDB 1N68) and *T. thermophilus* Tth (PDB 2XU9), is shown in dark purple and pink, respectively[10]. **(b)** Clustered heat map showing a color palette where dark blue and light yellow correspond to the highest and lowest distances, respectively, between MCOs cavities.

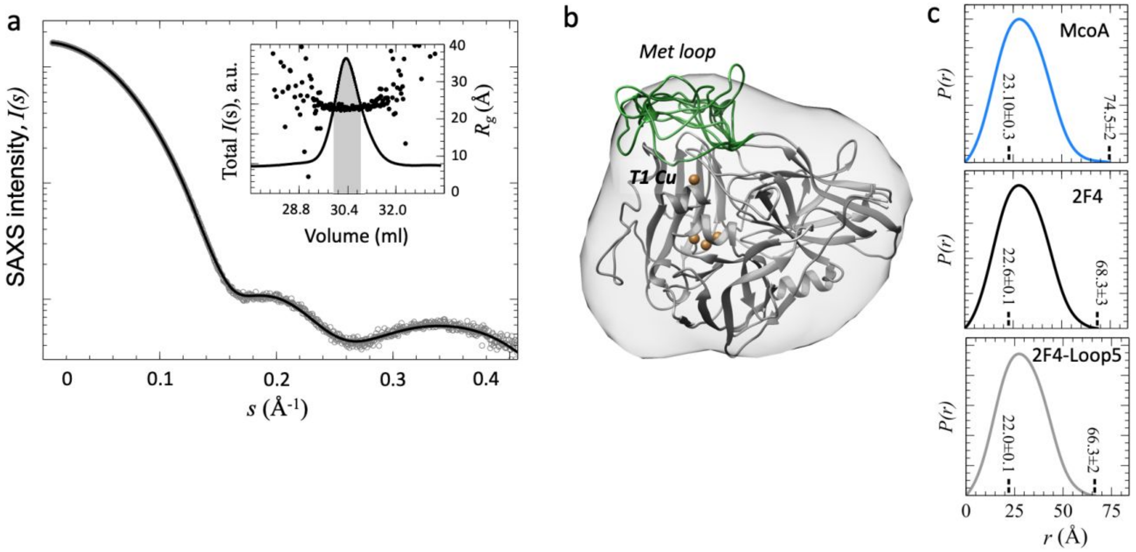

Figure S4. (**a**) **SEC-SAXS profile for 2F4**. This was obtained by integrating the central region of the monomeric single-peak eluted from a 24 ml Superdex-200 column. The inset shows total scattering intensity I(s), over the entire s range, from each frame acquired along elution volume and respective Rg-value (black circles). The flat variation of Rg reflects a pure monodisperse sample. (**b**) Ab initio model from the SAXS-data (gray surface envelope) well encapsulating a representative sub-ensemble of the closed-state. For reference, the Met-loop dispositions, on top of the T1 Cu, are in green. **(c)** Normalized pair-distance distribution P(r) of each SEC-purified McoA-wild-type (blue), 2F4 (black) and 2F4-Loop5 (grey). Note that 2F4 has smaller overall size parameters (Rg, Dmax) when compared to wild-type McoA [1], more akin to those of the truncated variant 2F4-Loop5. The derived Rg and Dmax values are in dashed lines (more detail on Table S3).

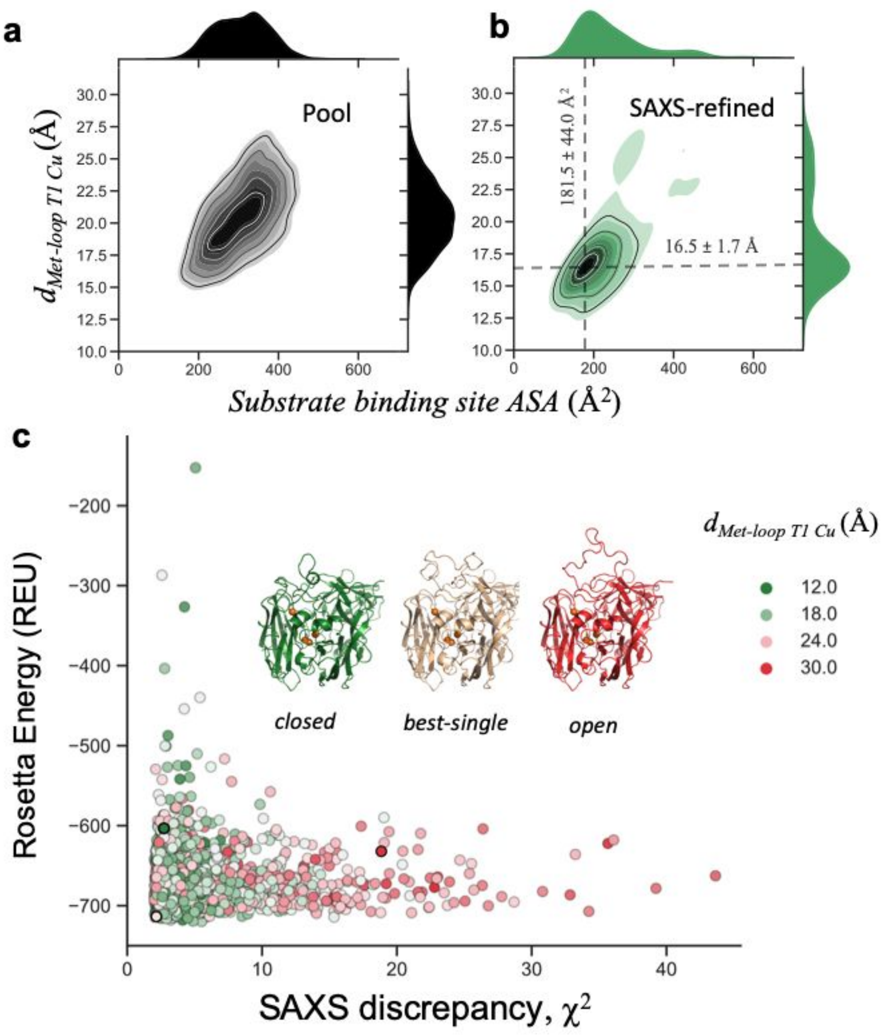

Figure S5. Met-loop Modeling (a) 2D kernel density of the initial pool of loop models created with Rosetta (gray-palette contours) and **(b)** EOM-refined ensembles of Met-loop in 2F4 (green-palette contours) plotted in the $\mathbf{d}_{\text{Met-loop-T1Cu}}$ and substrate binding site ASA conformational space. Solely one family of structures is observed for the refined-ensemble. This single-cluster is centered at 16.5 ± 1.7 Å and 181.5 ± 44.0 Å^2 and composed by relatively more compact Met-loop states. **(c)** Rosetta energy vs. SAXS discrepancy, χ^2 scatter plot for 2500 loop reconstructions on 2F4 X-ray structure as the template. REU stands for Rosetta Energy Units. This scatter-plot shows all structures' energies and their SAXS discrepancy scores (reduced χ2), colored based on the distance between the Met-loop and T1 Cu. The circles with black borders are the closed (green), open (red), and best-scored (yellow) models that we used as starting structures in MD simulations (see Fig. S6). The inset shows their ribbon representations in the same color code.

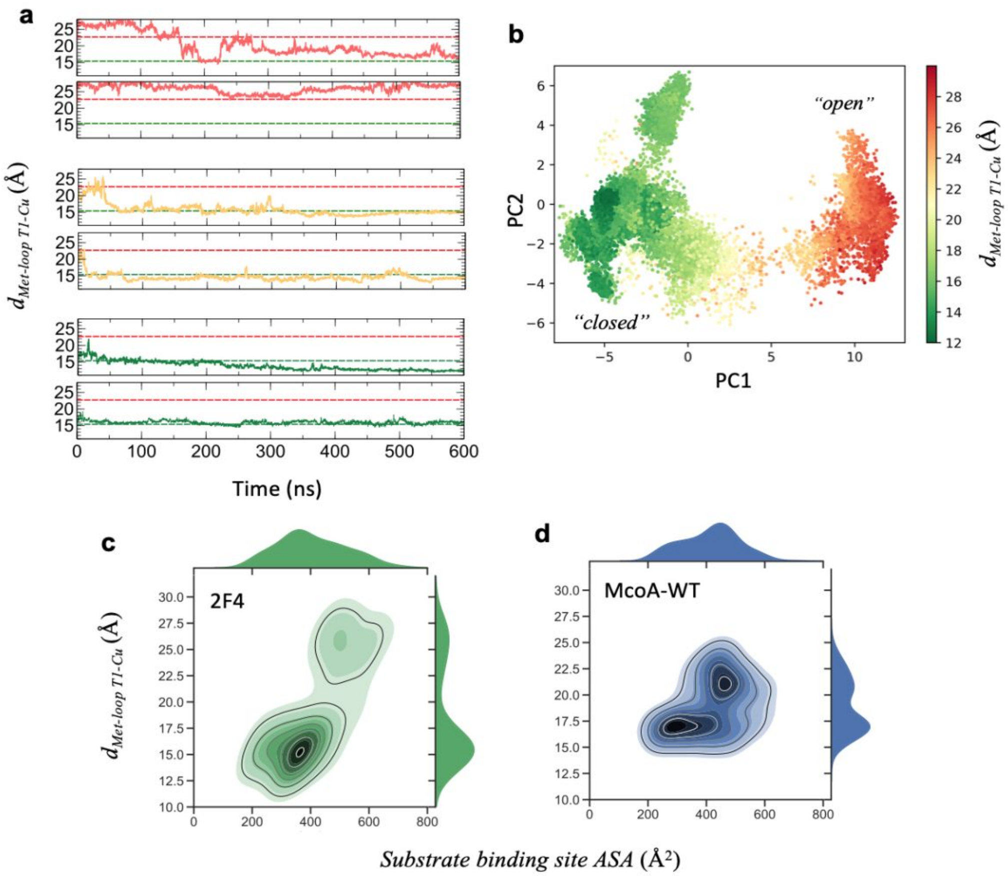

Figure S6. Met-loop from molecular dynamic simulations (**a**) Time evolution of the distance between T1 Cu and the Met-loop during MD simulations. Each panel displays two MD simulations of 2F4 (2x600ns) starting from three different loop models: an open (red), the best-single state (yellow), and a closed (green) SAXS-driven conformer (see Fig. S5). The Met-loop adopts distances to the T1-Cu ($d_{\text{Metloop-T1 Cu}}$) stable over time, predominantly fluctuating near the closed-like sates (green dashed line) states. Except for one trajectory, we observed noticeable open-to-closed transitions. (**b**) Principal component analysis (PCA) on 2F4 Molecular Dynamics. Each point, colored according to the distance between the T1 Cu and the center of mass of the Met-rich loop, represents a structural snap-shot of the 3.6 μs simulation projected onto the two most significant components (PC1 and PC2) that describe conformational variability, which here corresponds to open and closed conformation of the Met-loop. Along the simulations, 2F4 predominantly adopts a closed conformation. **(c-d)** Met-loop plotted in the dMet-loop-T1Cu and substrate binding site ASA conformational space for 2F4 (green-contours) and McoA (blue-contours) trajectories. In the 2F4, Met-loop explores mostly closed structures clustered in one prominent peak, whereas McoA[1] has two well-defined families of conformations reminiscent to their SAXS-ensembles.

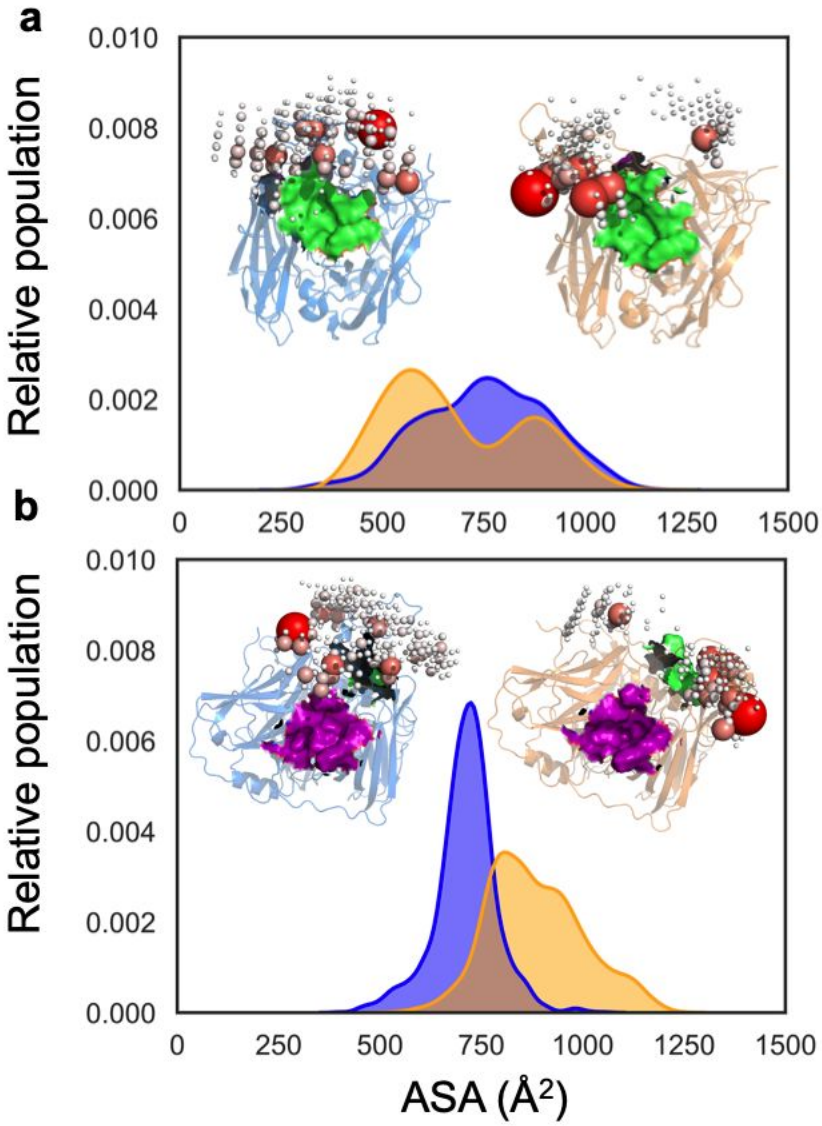

Figure S7. Cavities size and accessibility. Distribution of the accessible solvent surface area for cavity A (**a**, green) and cavity B (**b**, purple) in McoA (blue) and 2F4 (orange) from the simulations. Met-loop sampling over the T1 Cu is displayed as defined in Fig. 3**.** In 2F4, the flexible Met-loop closes over T1 Cu in the vicinity of cavity A, with cavity B widely accessible for potential binding.

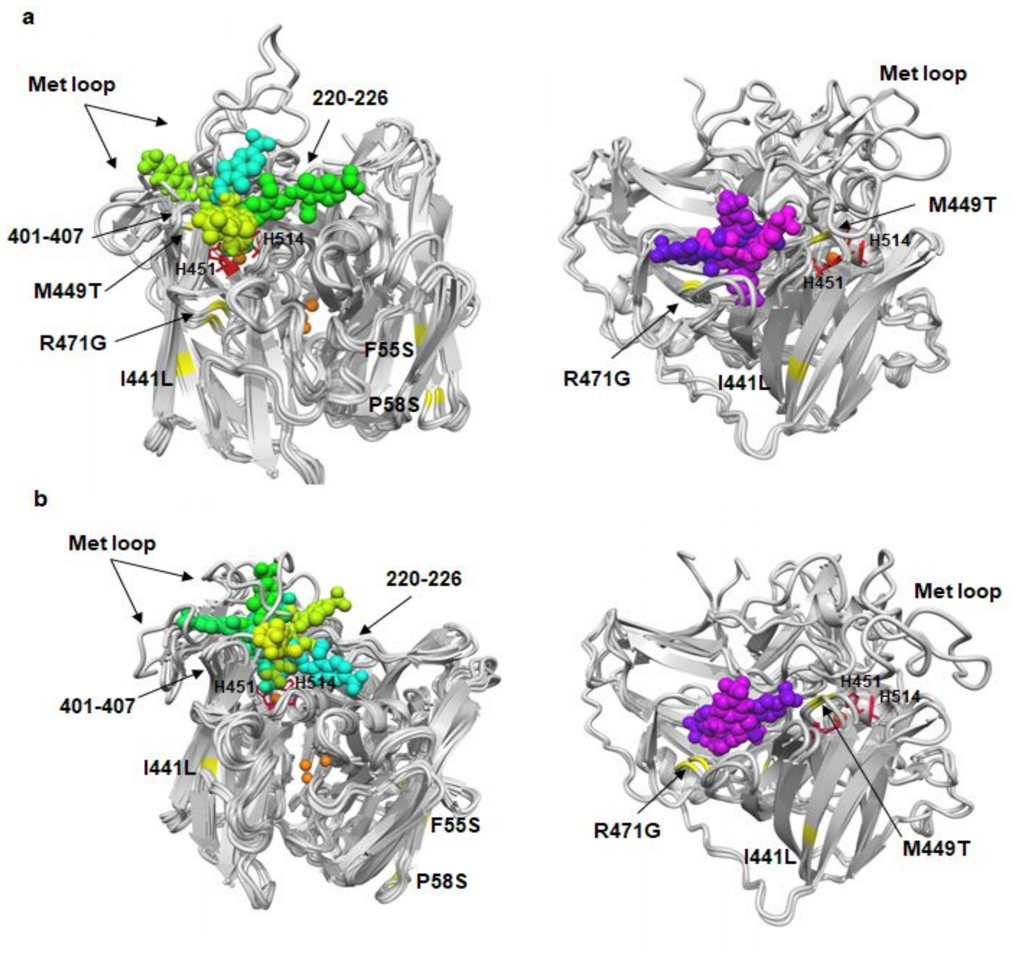

Figure S8. Binding of ABTS to wild-type and 2F4. Molecular representation of ABTS binding in the proximity of the T1 Cu for (**a**) wild-type and (**b**) 2F4. An ensemble of 1998 protein structures taken from the MD simulations was used in each case. Representative sub-ensembles of bound ABTS molecules are shown in green (cavity A) and purple (cavity B) colored balls. The protein backbone is depicted in grey cartoon, with the mutated positions marked in yellow. Copper atoms are depicted as orange spheres and histidines coordinating T1 Cu in red sticks. Binding to cavity A involves different orientations. Overall binding modes are similar for wild-type and 2F4.

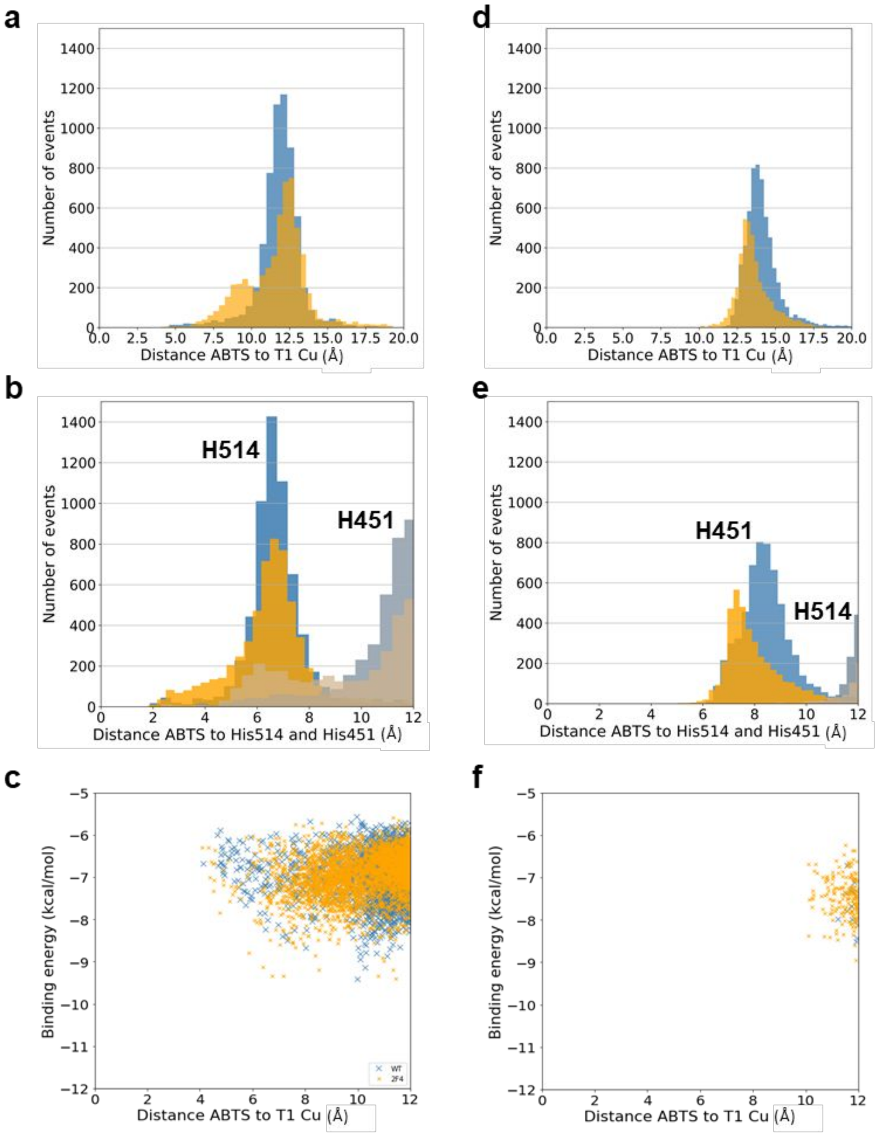

Figure S9. Local dockings of ABTS to wild-type and 2F4. Full histograms of ABTS binding distances (in Å) to T1 Cu, to T1 Cu coordinating His and binding energy (in kcal/mol) as a function of the ABTS to T1 Cu distance, are given for binding at (**a, b, c**) cavity A and (**d, e, f**) cavity B. Data is represented in blue and gray for wild-type and orange and tan for 2F4. For cavity A, ABTS can get significantly closer to the T1 Cu in 2F4 as compared to wild-type, whereas ABTS binding to cavity B give similar results for 2F4 and wild-type, with slightly shorter ABTS to T1 Cu distances in 2F4. Binding to cavity A produces shorter distances (2.5 – 8.0 Å) of ABTS to H514 as compared to H451 (side-chains), suggesting that electron transfer to Cu1 could go through the H514 coordinating residue. Distances are shorter in the case of 2F4. For cavity B, ABTS is located at shorter distances to H451 (starting at ~ 5/6 Å) as compared to H514, suggesting that electron transfer to Cu1 could go through the H451 T1 Cu coordinating residue.

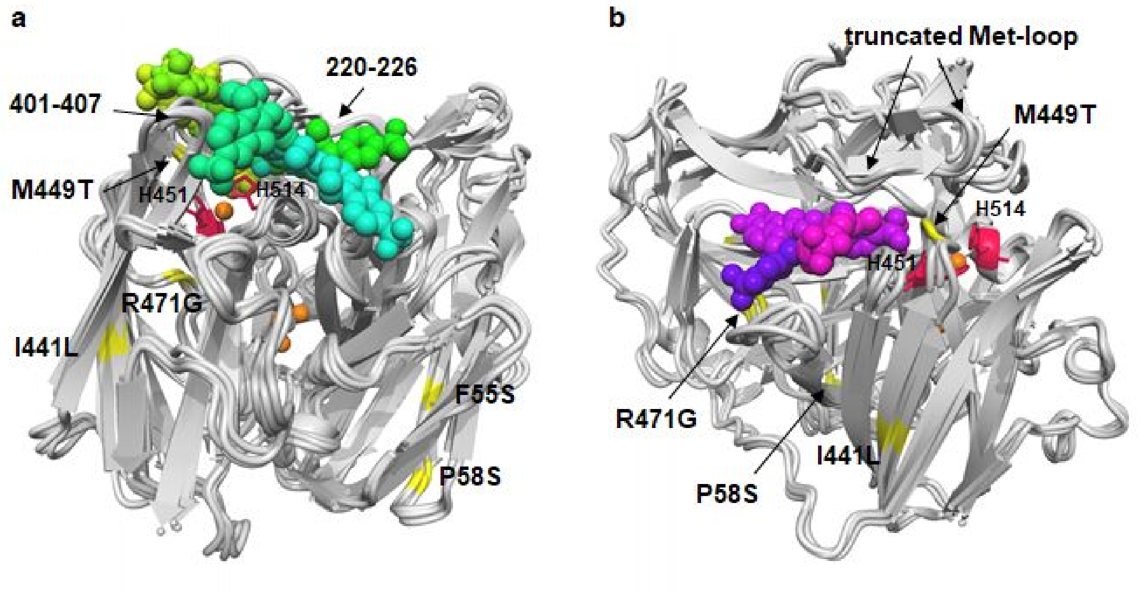

Figure S10. Docking of ABTS to cavity A and cavity B of Met-loop truncated variants. An ensemble of 400 protein structures taken from the MD simulations was used in each case. Molecular representation of ABTS binding in the proximity of the T1 Cu: cavity A for wild-type **(a)** and cavity B for 2F4 **(b)**. The protein backbone is depicted in grey cartoon, with the mutated positions marked in yellow, and representative of sub-ensembles of ABTS molecules in colored balls (green for cavity A and purple for cavity B). Copper atoms are depicted as orange spheres and histidines coordinating T1 Cu in red sticks. wild-type numbering of residues is used for better comparison.

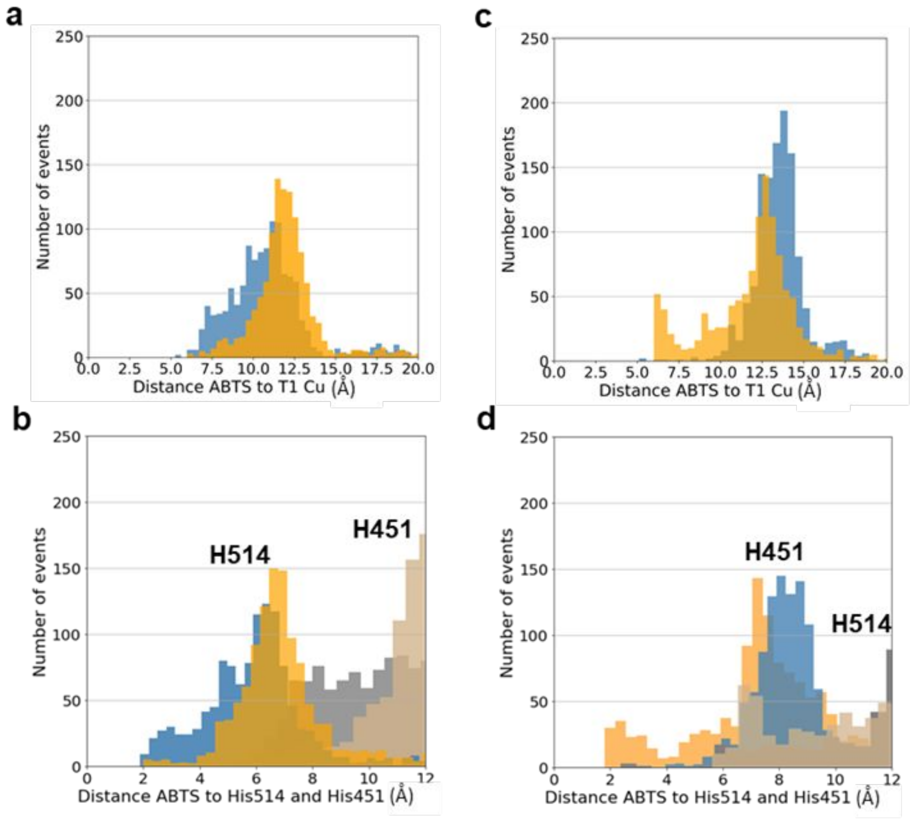

Figure S11. Local docking of ABTS to cavity A (a,b) and B (c,d) of the loop-truncated variants (loop 5). Full histograms of ABTS to T1 Cu, to H451 and to H514 distances (in Å) for cavity A and cavity B. Wild-type numbering of residues is used for better comparison. Data is represented in blue and gray for wild-type and in orange and tan for 2F4.

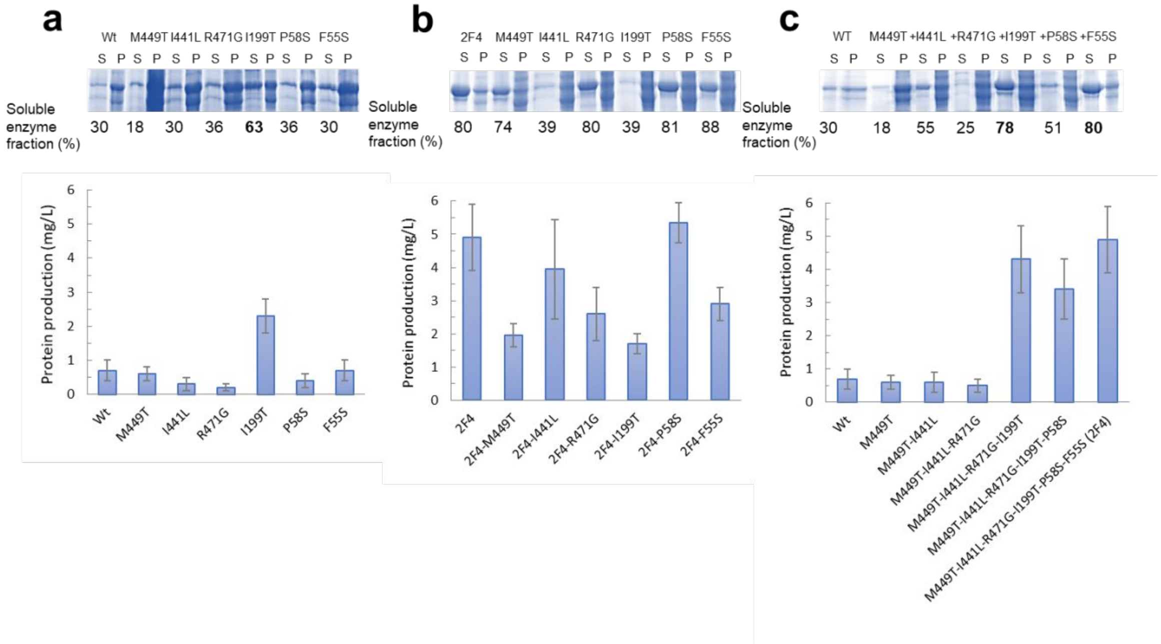

Figure S12. Soluble protein fractions and production yields of variants: after single addition to wild-type **(a)**, single removal from 2F4 background **(b)**, sequential addition to wild-type **(c)** of mutations M449T, I441L, R471G, I199T, P58S and F55S. The supernatant and insoluble pellet fractions of the partially purified crude extracts (after heating at 80°C) (S and P, respectively) were analyzed by SDS-PAGE, and the percentage of enzyme in the soluble fraction was determined by the relative intensities of the supernatant and pellet bands.

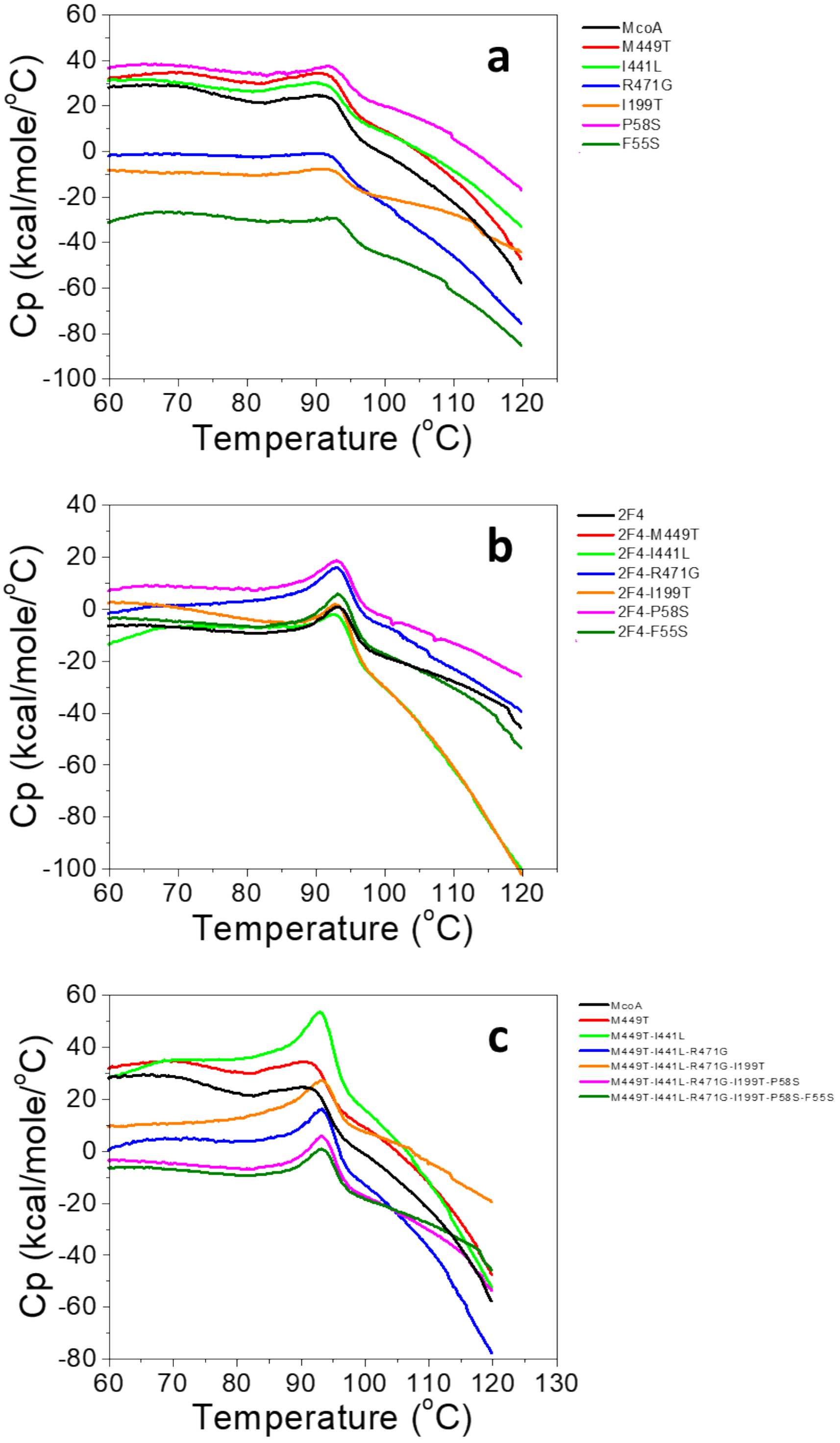

Figure S13. Excess heat capacity of wild type and variants constructed by site-directed mutagenesis obtained from differential scanning calorimetry.

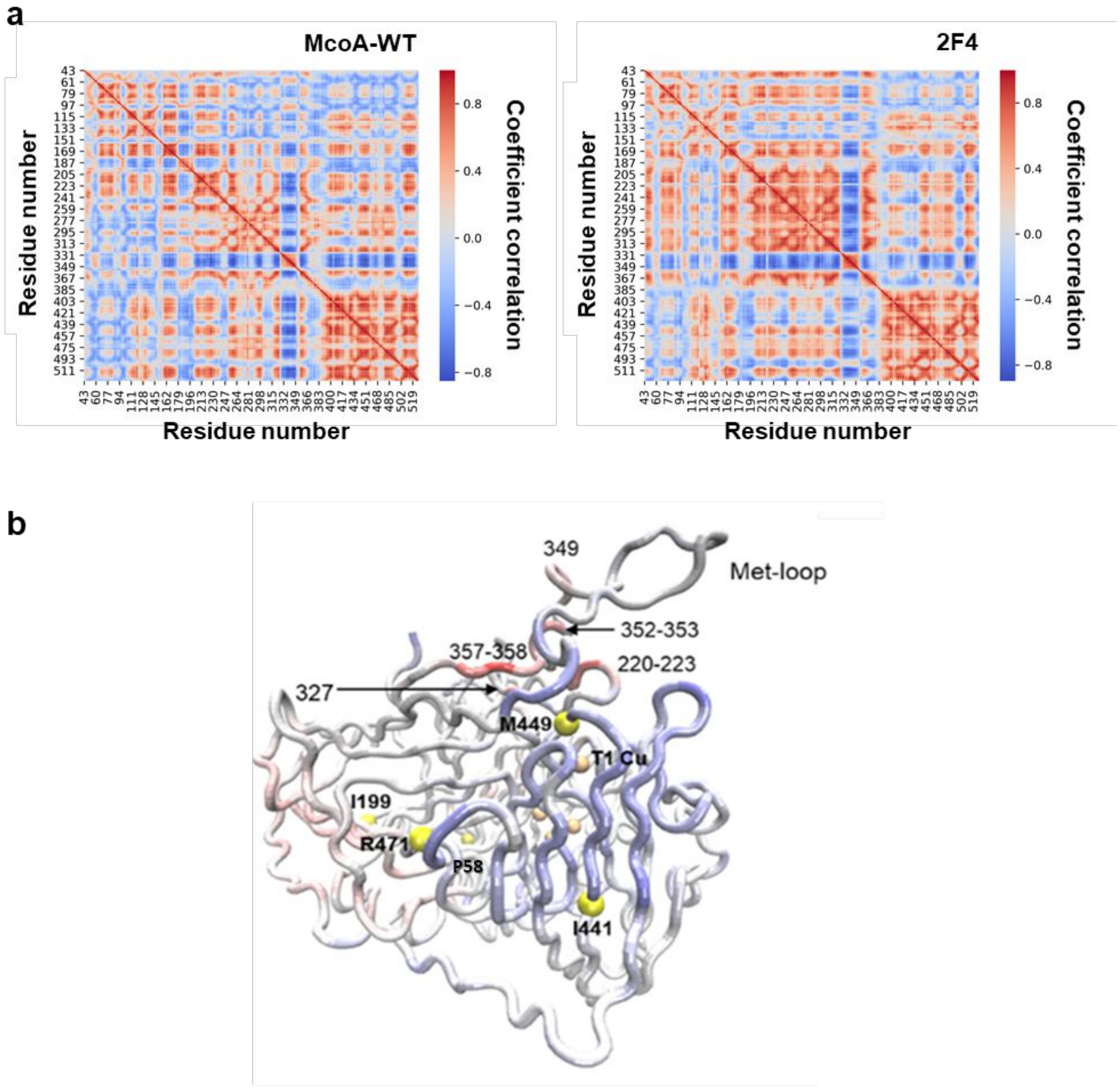

Figure S14. (a) Dynamical cross-correlation (DCC) maps for wild-type (left) and 2F4 (right). (**b**) The absolute difference of the per-residue averaged values is plotted onto the molecular representation of McoA (blue, white to red indicates higher values). Mutated positions and Cu atoms are yellow and orange spheres, respectively. The main changes are in the Met-loop base (residues 357 and 358) and its edge residues 352, 349, and 327, and the 220-223 fragment belonging to the loop 220-226. Other areas comprise protein regions located on the opposite side of the T1 Cu centre.

REFERENCES

1. Borges, P. T.; Brissos, V.; Hernandez, G.; Masgrau, L.; Lucas, M. F.; Monza, E.; Frazao, F.; Cordeiro, T. N.; Martins, L. O., The Methionine-Rich Loop of Multicopper Oxidase McoA Follows Open-To-Close Transitions with a Role in Catalysis. *ACS Catal* **2020,** *10*, 7162-7176.
2. Fernandes, A. T.; Soares, C. M.; Pereira, M. M.; Huber, R.; Grass, G.; Martins, L. O., A Robust Metallo-Oxidase from the Hyperthermophilic Bacterium *Aquifex aeolicus*. *FEBS J* **2007,** *274*, 2683-2694.
3. Brissos, V.; Ferreira, M.; Grass, G.; Martins, L. O., Turning A Hyperthermostable Metallo-Oxidase into a Laccase by Directed Evolution. *ACS Catal* **2015,** *5*, 4932-4941.
4. Karplus, P. A.; Diederichs, K., Linking Crystallographic Model and Data Quality. *Science* **2012,** *336* (6084), 1030-1033.
5. Arndt, U. W.; Crowther, R. A.; Mallett, J. F. W., A Computer-Linked Cathode-Ray Tube Microdensitometer for X-Ray Crystallography. *J Phys E Sci Instrum* **1968,** *1* (5), 510-516.
6. Diederichs, K.; Karplus, P. A., Improved R-Factors For Diffraction Data Analysis In Macromolecular Crystallography. *Nat. Struct. Biol.* **1997,** *4*, 269-275.
7. Weiss, M. S., Global indicators of X-ray data quality. *J. Appl. Crystallogr.* **2001,** *34*, 130-135.
8. Piiadov, V.; de Araujo, E. A.; Neto, M. O.; Craievich, A. F.; Polikarpov, I., SAXSMoW 2.0: Online Calculator of the Molecular Weight of Proteins In Dilute Solution from Experimental SAXS Data Measured On A Relative Scale. *Protein Sci.* **2019,** *28*, 454-463.
9. Franke, D.; Petoukhov, M. V.; Konarev, P. V.; Panjkovich, A.; Tuukkanen, A.; Mertens, H. D. T.; Kikhney, A. G.; Hajizadeh, N. R.; Franklin, J. M.; Jeffries, C. M.; Svergun, D. I., ATSAS 2.8: A Comprehensive Data Analysis Suite for Small-Angle Scattering from Macromolecular Solutions. *J Appl Crystallogr* **2017,** *50*, 1212-1225.
10. Bello, M.; Correa-Basurto, J.; Rudino-Pinera, E., Simulation of the Cavity-Binding Site of Three Bacterial Multicopper Oxidases Upon Complex Stabilization: Interactional Profile and Electron Transference Pathways. *J Biomol Struct Dyn* **2014,** *32*, 1303-17.
